# Short-Chain Fatty Acid-Producing Gut Microbiota Is Decreased in Parkinson’s Disease but Not in Rapid-Eye-Movement Sleep Behavior Disorder

**DOI:** 10.1128/mSystems.00797-20

**Published:** 2020-12-08

**Authors:** Hiroshi Nishiwaki, Tomonari Hamaguchi, Mikako Ito, Tomohiro Ishida, Tetsuya Maeda, Kenichi Kashihara, Yoshio Tsuboi, Jun Ueyama, Teppei Shimamura, Hiroshi Mori, Ken Kurokawa, Masahisa Katsuno, Masaaki Hirayama, Kinji Ohno

**Affiliations:** a Division of Neurogenetics, Center for Neurological Diseases and Cancer, Nagoya University Graduate School of Medicine, Nagoya, Japan; b Department of Pathophysiological Laboratory Sciences, Nagoya University Graduate School of Medicine, Nagoya, Japan; c Division of Neurology and Gerontology, Department of Internal Medicine, School of Medicine, Iwate Medical University, Iwate, Japan; d Department of Neurology, Okayama Kyokuto Hospital, Okayama, Japan; e Department of Neurology, Fukuoka University, Fukuoka, Japan; f Division of Systems Biology, Center for Neurological Diseases and Cancer, Nagoya University Graduate School of Medicine, Nagoya, Japan; g Genome Evolution Laboratory, Department of Informatics, National Institute of Genetics, Mishima, Japan; h Department of Neurology, Nagoya University Graduate School of Medicine, Nagoya, Japan; Vall d’Hebron Research Institute (Ed. Mediterranea)

**Keywords:** rapid-eye-movement behavior disorder, gut microbiota, meta-analysis, Parkinson’s disease, topic model

## Abstract

Gut dysbiosis has been repeatedly reported in Parkinson’s disease (PD) but only once in idiopathic rapid-eye-movement sleep behavior disorder (iRBD) from Germany. Abnormal aggregation of α-synuclein fibrils causing PD possibly starts from the intestine, although this is still currently under debate. iRBD patients frequently develop PD. Early-stage gut dysbiosis that is causally associated with PD is thus expected to be observed in iRBD. We analyzed gut microbiota in 26 iRBD patients and 137 controls by 16S rRNA sequencing (16S rRNA-seq). Our iRBD data set was meta-analyzed with the German iRBD data set and was compared with gut microbiota in 223 PD patients. Unsupervised clustering of gut microbiota by LIGER, a topic model-based tool for single-cell RNA sequencing (RNA-seq) analysis, revealed four enterotypes in controls, iRBD, and PD. Short-chain fatty acid (SCFA)-producing bacteria were conserved in an enterotype observed in controls and iRBD, whereas they were less conserved in enterotypes observed in PD. Genus *Akkermansia* and family *Akkermansiaceae* were consistently increased in both iRBD in two countries and PD in five countries. Short-chain fatty acid (SCFA)-producing bacteria were not significantly decreased in iRBD in two countries. In contrast, we previously reported that recognized or putative SCFA-producing genera *Faecalibacterium*, *Roseburia*, and *Lachnospiraceae ND3007 group* were consistently decreased in PD in five countries. In α-synucleinopathy, increase of mucin-layer-degrading genus *Akkermansia* is observed at the stage of iRBD, whereas decrease of SCFA-producing genera becomes obvious with development of PD.

**IMPORTANCE** Twenty studies on gut microbiota in PD have been reported, whereas only one study has been reported on iRBD from Germany. iRBD has the highest likelihood ratio to develop PD. Our meta-analysis of iRBD in Japan and Germany revealed increased mucin-layer-degrading genus *Akkermansia* in iRBD. Genus *Akkermansia* may increase the intestinal permeability, as we previously observed in PD patients, and may make the intestinal neural plexus exposed to oxidative stress, which can lead to abnormal aggregation of prion-like α-synuclein fibrils in the intestine. In contrast to PD, SCFA-producing bacteria were not decreased in iRBD. As SCFA induces regulatory T (Treg) cells, a decrease of SCFA-producing bacteria may be a prerequisite for the development of PD. We propose that prebiotic and/or probiotic therapeutic strategies to increase the intestinal mucin layer and to increase intestinal SCFA potentially retard the development of iRBD and PD.

## Abstract (precis)

Twenty studies on gut microbiota in PD have been reported, whereas only one study has been reported on iRBD from Germany. iRBD has the highest likelihood ratio to develop PD. Our meta-analysis of iRBD in Japan and Germany revealed increased mucin-layer-degrading genus *Akkermansia* in iRBD.

## INTRODUCTION

Parkinson’s disease (PD) is a progressive neurodegenerative disease exhibiting four major motor deficits of tremor, slowness of movement, rigidity, and postural instability (1). PD also exhibits nonmotor symptoms that are characterized by dysautonomia (constipation, vomiting, orthostatic hypotension, abnormal sweating, and dysuria) and mental disorders (depression, anxiety disorder, visual hallucination, and dementia) (1). Turning our eyes to the pathophysiology of PD, motor symptoms of PD are caused by loss of the dopaminergic neurons in the substantia nigra. On the other hand, nonmotor symptoms of PD are caused by loss of neurons in the other brain regions (the locus coeruleus, nucleus basalis of Meynert, pedunculopontine nucleus, raphe nucleus, dorsal motor nucleus of the vagus, amygdala, and hypothalamus) affecting nondopaminergic neurotransmitter systems (the cholinergic, adenosinergic, glutamatergic, GABAergic, noradrenergic, serotonergic, and histaminergic systems) (2–4). Loss of the dopaminergic or nondopaminergic neurons in various brain regions is mostly accounted for by abnormally aggregated α-synuclein fibrils (Lewy bodies) in the neuronal cells. In addition, oxidative stress (5, 6), autophagy dysfunction (7, 8), proteostasis failure (9–11), vesicular trafficking defects (12–14), and neuroinflammation (15–17) trigger loss of these neurons. Abnormal aggregation of α-synuclein fibrils behaves like prions and is propagated to other neuronal cells probably via synapses (18). Lewy bodies are also observed in the cerebral cortex, the olfactory bulb (19), the autonomic nervous system (20), the salivary glands (21), the skin (22), and the intestine (21, 23, 24). Abnormal aggregation of α-synuclein fibrils possibly starts in the intestinal neural plexus and ascends to the substantia nigra in most PD patients, although 7 to 11% of PD patients have Lewy bodies in the brain but not in the dorsal motor nucleus of the vagus (19, 25–30). Constipation, rapid-eye-movement sleep behavior disorder (RBD), and depression are frequently predisposed to the development of motor symptoms in PD in this order, which is in accordance with the ascending α-synucleinopathy (1). A total of 20 studies had been reported by us (31, 32) and others (33–50) on gut microbiota in PD. Our recent report included the largest cohort of PD patients and the development of a novel nonparametric meta-analysis method that was applied to analyze gut microbiota in PD in five countries (32). Our meta-analysis revealed that increased genus *Akkermansia* and decreased genera *Roseburia* and *Faecalibacterium* were shared in PD across countries. In addition, these taxonomic changes were independent of the confounding effects of constipation, body mass index (BMI), sex, age, and catechol-O-methyl transferase (COMT) inhibitor intake.

RBD is characterized by dream-enactment behaviors during the rapid-eye-movement sleep, when normal people lose muscle tone, called a state of atonia (51). RBD is categorized into idiopathic RBD (iRBD) and symptomatic RBD. The prevalence of iRBD is estimated to be 0.5 to 2% (52, 53). iRBD frequently predisposes to neurodegenerative α-synucleinopathies including PD, dementia with Lewy bodies (DLB), and multiple system atrophy (MSA) (51). iRBD patients sometimes have subtle sensory, motor, and cognitive deficits, as well as constipation, before the onset of PD and other α-synucleinopathies (51). PD has been classified into three groups according as the disease progresses: preclinical PD (no overt symptoms even in the presence of neurodegeneration), prodromal PD (overt symptoms but lacking the criteria of PD), and clinical PD (overt symptoms satisfying the criteria of PD) (54). iRBD is the most dependable hallmark of prodromal PD (54). Similarly, the likelihood ratio of iRBD to develop PD is as high as 130 (55). Thus, therapeutic intervention to prevent transition from iRBD to PD has a potential to become a causative treatment for PD (56).

In contrast to as many as 20 studies reported on gut microbiota in PD as stated above, only one study has been reported on 21 iRBD patients along with 76 PD patients from Germany (43). The authors reported that gut microbiota in iRBD was similar to that in PD. We recently reported increased genus *Akkermansia* and decreased short-chain fatty acid (SCFA)-producing taxa in PD in five countries including the German data set (32, 43). We here performed 16S rRNA sequencing (16S rRNA-seq) analysis of 26 iRBD patients and 137 controls. We also meta-analyzed our data set with the German data set using a nonparametric meta-analysis method that we developed previously to identify shared taxonomic changes between the two countries and compared iRBD-associated taxonomic changes in two countries with PD-associated changes in five countries.

## RESULTS

### Differences in demographic and clinical features, diet, and medications between controls and iRBD in our data set.

All iRBD patients were diagnosed according to the International Classification of Sleep Disorders Criteria-Third Edition (57). To search for possible confounding factors, we compared four features (age, sex, BMI, and constipation) between iRBD and controls in our data set (Table 1). Compared to controls, iRBD patients had higher ages and higher BMI and included more males. Similarly, the ratio of constipation was higher in iRBD patients. The Unified Parkinson’s Disease Rating Scale (UPDRS) and Mini-Mental Status Examination (MMSE) scores indicated lack of Parkinsonian symptoms and lack of cognitive deficits (Table 2). iRBD patients had similar autonomic dysfunctions as PD patients in our cohort (32) (Table 2). iRBD and PD patients took proton pump inhibitors (PPI) more frequently than controls (Table 3). PD patients drank coffee less frequently than controls (Table 3), as has been previously reported (58–60).

**TABLE 1:** Demographic and clinical features of iRBD and controls in our data set

	iRBD patients (*n* = 26)	Controls (*n* = 137)	*P* value^*c*^
Age (yr)^*a*^	74.5 ± 6.4	68.3 ± 9.8	*2.2E−3
Sex (males/females)^*b*^	20/6 (76.9% males)	62/71 (45.3% males)	*5.2E−3
Body mass index (BMI)^*a*^	24.4 ± 2.4	22.9 ± 3.1	*0.018
No. with constipation (less than or equal to twice a wk)^*b*^	9 (34.6%)	6 (4.4%)	*5.7E−5

a Mean and SD are indicated, and Student’s *t* test is applied.

b Fisher’s exact test is applied.

c *, *P* value < 0.05.

**TABLE 2:** Clinical features of iRBD and PD in our data set

	iRBD patients	PD patients (32)	*P* value^*c*^
Total UPDRS^*a*^	7.6 ± 5.5 (*n *= 17) (range, 2 to 24)	50.1 ± 23.1 (*n *= 223) (range, 0 to 153)	*2.2E−12
UPDRS III^*a*^	2.0 ± 2.6 (*n *= 17) (range, 0 to 9)	26.4 ± 13.5 (*n *= 223) (range, 0 to 84)	*2.3E−12
MMSE^*a*^	28.2 ± 1.9 (*n *= 17) (range, 22 to 30)	28.0 ± 2.5 (*n *= 223) (range, 15 to 30)	0.81
Duration (yr)^*a*^	6.4 ± 4.8 (*n *= 26) (min 0.1, max 20)	7.5 ± 6.1 (*n *= 223) (min 0.2, max 42)	0.38
No. with constipation (less than or equal to twice a wk)^*b*^	9/17 (34.6%)	80/139 (36.5%)	1.0
No. with fecal incontinence^*b*^	2/24 (7.7%)	18/205 (8.1%)	1.0
No. with nocturia^*b*^	19/7 (73.1%)	145/78 (65.0%)	0.51
No. with orthostatic hypotension^*b*^	7/19 (26.9%)	58/164 (26.1%)	1.0
No. with abnormal sweating^*b*^	3/23 (11.5%)	52/170 (23.4%)	0.22

a Mean and SD are indicated, and Student’s *t* test is applied.

b The numbers indicate positive cases/negative cases (ratio of positive cases). Fisher’s exact test is applied. Note that total UPDRS and UPDRS III scores are not zero in iRBD patients but are similar to those reported in healthy aged individuals (93).

c *, P value < 0.05.

**TABLE 3:** Medications and diet in controls, iRBD patients, and PD patients in our data set

No. with medication or diet	Controls	iRBD patients	PD patients	*P* value (controls vs iRBD)^*d*^	*P* value (controls, iRBD, vs PD)^*d*^
Proton pump inhibitor^*a*^	13/124 (9.5%)	7/19 (26.9%)	35/188 (15.7%)	*0.021	*0.040
H_2_ blocker^*a*^	5/132 (3.6%)	1/25 (3.8%)	8/215 (3.6%)	1.00	1.00
Antihyperlipidemic drug^*a*^	28/109 (13.1%)	4/22 (15.4%)	31/192 (13.9%)	0.79	0.26
Angiotensin II receptor blocker^*a*^	20/117 (14.6%)	6/20 (23.1%)	24/199 (10.8%)	0.18	0.22
Calcium channel blocker^*a*^	34/103 (24.8%)	8/18 (30.7%)	38/185 (17.0%)	0.62	0.088
Rice^*b*^	0 (0%), 4 (3.0%), 131 (97.0%)	0 (0%), 1 (3.8%), 25 (96.2%)	0 (0%), 6 (2.7%), 213 (97.3)	0.59	0.79
Bread^*b*^	13 (9.6%), 45 (33.3%), 77 (57.0%)	0 (0%), 8 (30.8%), 18 (69.2%)	20 (9.1%), 77 (35.2%), 122 (55.7%)	0.22	0.53
Noodles^*b*^	13 (9.7%), 86 (64.2%), 35 (26.1%)	2 (7.7%), 17 (65.3%), 7 (26.9%)	25 (11.4%), 148 (67.6%), 46 (21.0%)	1.00	0.80
Potatoes^*b*^	6 (4.5%), 68 (50.7%), 60 (44.8%)	3 (11.5%), 13 (50.0%), 10 (38.5%)	11 (5.0%), 118 (53.9%), 90 (41.1%)	0.32	0.59
Seafood^*b*^	1 (0.7%), 38 (28.4%), 95 (70.9%)	0 (0%), 12 (46.2%), 14 (53.8%)	0 (0%), 87 (39.7%), 132 (60.3%)	0.25	0.060
Meat^*b*^	4 (3.0%), 32 (24.1%), 97 (72.9%)	1 (3.8%), 10 (38.5%), 15 (57.7%)	3 (1.4%), 68 (31.1%), 148 (67.6%)	0.20	0.23
Milk^*b*^	39 (29.1%), 21 (15.7%), 74 (55.2%)	8 (30.8%), 7 (26.9%), 11 (42.3%)	60 (27.4%), 48 (21.9%), 111 (50.7%)	0.32	0.49
Dairy food^*b*^	29 (21.6%), 26 (19.4%), 79 (59.0%)	6 (23.1%), 2 (7.7%), 18 (69.2%)	37 (16.9%), 45 (20.5%), 137 (62.6%)	0.37	0.44
Beans^*b*^	4 (3.0%), 34 (25.3%), 96 (71.6%)	3 (11.5%), 9 (34.6%), 14 (53.8%)	11 (5.0%), 66 (30.1%), 142 (64.8%)	0.066	0.21
Vegetables^*b*^	1 (0.74%), 8 (5.9%), 126 (93.3%)	0 (0%), 2 (7.7%), 24 (92.3%)	0 (0%), 26 (11.9%), 192 (88.0%)	0.72	0.19
Mannose-rich corm (konjac)^*b*^	28 (20.7%), 92 (68.1%), 15 (11.1%)	9 (34.6%), 16 (61.5%), 1 (3.8%)	54 (24.7%), 141 (64.3%), 24 (11.0%)	0.27	0.60
Mushroom^*b*^	7 (5.2%), 55 (40.7%), 73 (54.1%)	1 (3.8%), 14 (53.8%), 11 (42.3%)	19 (8.7%), 103 (47.0%), 97 (44.3%)	0.45	0.33
Seaweed^*b*^	10 (7.4%), 65 (48.1%), 60 (44.4%)	4 (15.4%), 13 (50.0%), 9 (34.6%)	20 (9.1%), 111 (50.7%), 88 (40.2%)	0.32	0.65
Coffee^*b*^	14 (10.3%), 18 (13.3%), 103 (76.3%)	3 (11.5%), 2 (7.7%), 21 (80.8%)	48 (22.0%), 50 (22.9%), 120 (55.0%)	0.81	*4.4E−3
Tea^*b*^	7 (5.2%), 17 (12.6%), 111 (82.2%)	4 (15.4%), 5 (19.2%), 17 (65.3%)	25 (11.6%), 29 (13.5%), 161 (74.9%)	0.071	0.13
Beer^*c*^	101 (75.4%), 24 (17.9%), 9 (6.7%)	18 (69.2%), 8 (30.8%), 0 (0%)	182 (82.0%), 35 (15.8%), 5 (2.3%)	0.18	0.073
Alcohol other than beer^*c*^	87 (64.9%), 10 (7.5%), 37 (27.6%)	17 (65.4%), 1 (3.8%), 8 (30.8%)	164 (73.5%), 13 (5.8%), 46 (20.6%)	0.89	0.41

a Positive cases/negative cases (ratio of positive cases).

b The first, second, and third items indicate the number of cases (ratio) who take the indicated food zero times a week, once or twice a week, and three or more times a week, respectively.

c The first, second, and third items indicate the number of cases (ratio) who take the indicated alcohol zero times a week, once to three times a week, and four or more times a week, respectively.

d *P* values are calculated by Fisher’s exact test. *, *P* value < 0.05.

### PERMANOVA to evaluate the differences in the overall composition of gut microbiota between controls and iRBD, as well as between iRBD and Hoehn and Yahr 1 scale of PD.

We performed 16S rRNA-seq analysis of gut microbiota in 26 patients with iRBD and 137 healthy controls. Permutational multivariate analysis of variance (PERMANOVA) compares the overall bacterial compositions between two groups by either taking into account or not the effects of possible confounding factors (61). PERMANOVA manages the effect of controls versus iRBD, and the effects of possible confounding factors, at an equal level. We first compared the overall bacterial compositions between controls and iRBD without considering possible confounding factors and found that the overall bacterial compositions were statistically different by all three distance metrics (Table 4A). PERMANOVA including the possible confounding factors showed that the difference was not accounted for by sex, BMI, constipation, or PPI (Table 4B). Age had an equivocal effect with one significant and two nonsignificant distance metrics (Table 4B). We next compared the overall bacterial compositions between iRBD and Hoehn and Yahr 1 of PD without considering possible confounding factors and found that the overall bacterial compositions were statistically different by two distance metrics (Table 4C). PERMANOVA including the possible confounding factors showed that the difference was not accounted for by age, sex, BMI, constipation, or PPI (Table 4D). Although these possible confounding factors had no essential effect on the overall bacterial composition, we took into account the effects of these possible confounding factors on individual genera and families in the following analysis.

**TABLE 4:** PERMANOVA to examine the effect of each factor on the overall bacterial composition in our data set^*b*^

	No. of iRBD patients	No. of controls	*P* value (Chao)	*P* value (weighted UniFrac)	*P* value (unweighted UniFrac)
(A)	26	137			
iRBD vs controls			*4.3E−03	*0.010	*3.2E−03
(B)	26	133^*a*^			
iRBD vs controls			*3.6E−03	*0.011	*5.7E−03
Age			*0.012	0.45	0.14
Sex			0.14	0.8	0.44
BMI			0.49	0.53	0.48
Constipation			0.12	0.1	0.62
PPI			0.092	0.26	0.26

	No. of iRBD patients	No. of Hoehn and Yahr 1 scale	*P* value (Chao)	*P* value (weighted UniFrac)	*P* value (unweighted UniFrac)
(C)	26	30			
iRBD vs Hoehn Yahr 1 scale			*0.011	*4.6E−03	0.44
(D)	26	30			
iRBD vs Hoehn Yahr 1 scale			*0.046	*7.4E−03	0.48
Age			0.46	0.45	0.43
Sex			0.46	0.42	0.63
BMI			0.90	0.79	0.24
Constipation			0.52	0.46	0.85
PPI			0.80	0.75	0.39

a Four controls lacking demographic features were excluded from the analysis.

b *P* values of three distance metrics (Chao, unweighted UniFrac, and weighted UniFrac) by PERMANOVA are indicated. PERMANOVA was used to examine the effect of “iRBD vs controls” (A), and “iRBD vs Hoehn and Yahr 1 scale of PD” (C), on the overall microbial composition without considering possible confounding factors. The effects of “iRBD vs controls” (B) and “iRBD vs Hoehn and Yahr 1 scale of PD” (D) were evaluated in the presence of the effects of sex, age, BMI, constipation, and PPI by PERMANOVA. In table sections B and D, the six features were equally evaluated in PERMANOVA. *, *P* value < 0.05.

### PCoA plot to analyze the overall composition of gut microbiota in controls, iRBD, and PD.

**FIG 1:**
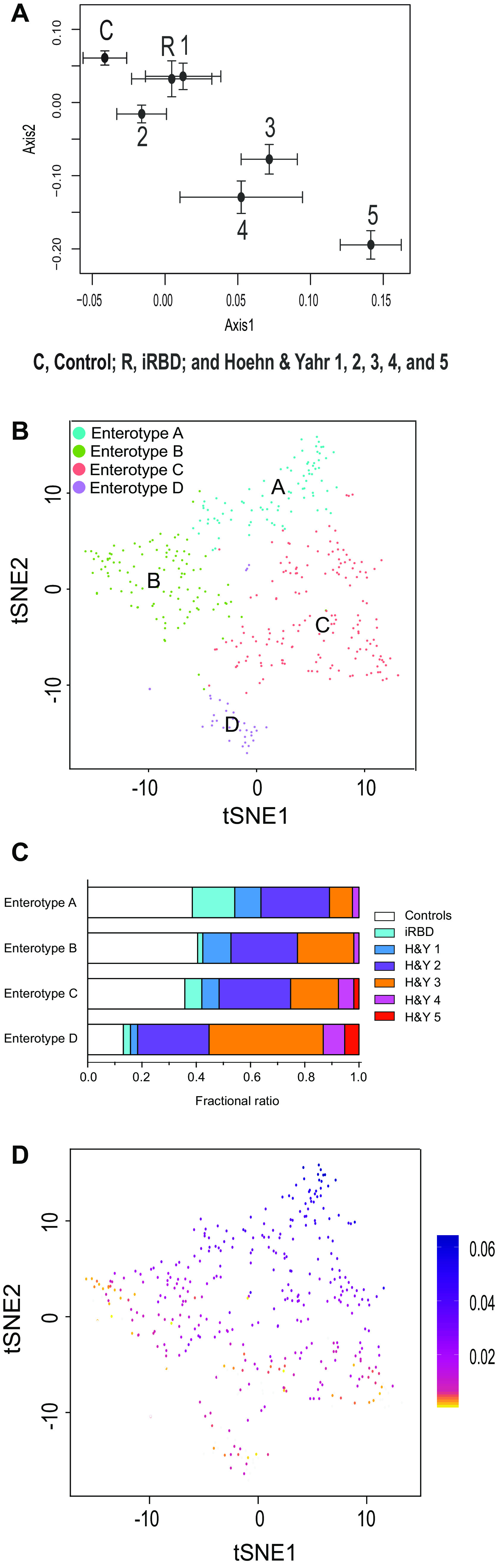
Overall compositions of gut microbiota in controls, iRBD, and PD (Hoehn and Yahr scales 1 to 5) in our data set. (A) PCoA plot showing the center of gravity of the overall compositions of gutmicrobiota in seven morbidity categories. The numbers of subjects in controls, iRBD, and Hoehn and Yahr scales 1 to 5 were 137, 26, 30, 99, 73, 16, and 5, respectively. Chao is used as a distance metric. Standard errors are indicated. (B) Unsupervised clustering of overall compositions of gut microbiota in controls, iRBD, and PD by LIGER yielded four enterotypes. t-Distributed stochastic neighbor embedding (tSNE) was adopted to visualize four clusters representing enterotypes A to D. (C) Fractional ratios of controls, iRBD, and Hoehn and Yahr (H&Y) scales 1 to 5 in each enterotype. (D) Bacterial abundances in a total of 386 subjects were factorized into multiple factors. The first factor is color-coded in each subject on a tSNE plot indicated in panel B. As SCFA-producing bacteria have high loadings in the first factor (see Table S1 in the supplemental material), individuals colored in blue carry a high proportion of SCFA-producing bacteria.

We conducted principal-coordinate analysis (PCoA) of gut microbiota in controls and iRBD in our data set, as well as gut microbiota in our previously reported PD subjects (Hoehn and Yahr scales 1 to 5) (32). The centers of gravity moved from the upper left to the lower right with disease progression from controls, to iRBD, to Hoehn and Yahr scales 1 to 5 (Fig. 1A). iRBD was positioned close to the mildest form of PD with Hoehn and Yahr scale 1.

10.1128/mSystems.00797-20.2 TABLE S1 The top 10 genera with the highest loadings in the first factor by LIGER analysis. Note that genus *Lachnospiraceae ND3007 group* is a putative SCFA producer, and the other nine genera are established SCFA producers. Download Table S1, DOCX file, 0.01 MB. Copyright © 2020 Nishiwaki et al. 2020 Nishiwaki et al. This content is distributed under the terms of the Creative Commons Attribution 4.0 International license.

### LIGER analysis to reveal unsupervised enterotypes in a combined data set of controls, iRBD, and PD.

We applied LIGER that was developed for topic model-based single-cell RNA sequencing (RNA-seq) analysis (62) to make unsupervised clustering of gut microbiota in controls, iRBD, and PD (Hoehn and Yahr scales 1 to 5). Each cluster should represent an enterotype. LIGER revealed four enterotypes A, B, C, and D (Fig. 1B). Examination of the proportion of controls, iRBD, and Hoehn and Yahr scales 1 to 5 in each enterotype revealed that the proportion of controls was decreased in the order of enterotypes A to D, while the proportions of Hoehn and Yahr scales 3 to 5 were increased in the same order (Fig. 1C). The proportion of iRBD was the highest in enterotype A. In factorization by LIGER, the first factor contributes most to differentiate enterotypes A to D, and genera with high loadings in the first factor are major determinants of enterotypes. Color-coding of the first factor in each subject showed a gradual decrease of the first factor from enterotypes A to D (Fig. 1D). The top 10 genera with the highest loadings in the first factor are indicated in Table S1 in the supplemental material. It was interesting that, among the 10 genera, nine produce SCFA and one putatively produces SCFA (*Lachnospiraceae ND3007 group*). Scatterplots of the 10 genera in each enterotype showed that the abundances of these genera were also decreased in the order of enterotypes A to D (see Fig. S1 in the supplemental material). Among the 10 genera, *Faecalibacterium*, *Roseburia*, and *Lachnospiraceae ND3007 group* were exactly the three genera that were decreased in PD in our previous meta-analysis of five countries (32). To summarize, unsupervised clustering of enterotypes revealed that enterotypes were shifted from A to D with transition from control, to iRBD, to Hoehn and Yahr scales 1 to 5 of PD and that SCFA-producing genera were decreased from enterotypes A to D.

10.1128/mSystems.00797-20.1 FIG S1 Relative abundances of 10 genera with the highest loadings in the first factor (Table S1) are plotted against enterotypes A to D generated by LIGER. Bar indicates the median value. *P* values of Jonckheere-Terpstra trend test are indicated in the upper right corner to show whether the genus increases or decreases monotonically. Download FIG S1, EPS file, 2.8 MB. Copyright © 2020 Nishiwaki et al. 2020 Nishiwaki et al. This content is distributed under the terms of the Creative Commons Attribution 4.0 International license.

### Analysis of each taxon between controls and iRBD in our data set.

We examined taxonomic differences between controls and iRBD in our data set using Analysis of Composition of Microbiomes (ANCOM) (63) and Wilcoxon rank sum test (Table S2a at the genus level and Table S2b at the family level in the supplemental material). ANCOM was developed to reduce false discoveries by exploiting microbial compositional constraints (63, 64). The analyses revealed that seven genera were increased in iRBD (*Ruminococcus 2*, *Alistipes*, *Akkermansia*, *Ruminococcaceae UCG-005*, *Ruminococcaceae UCG-004*, [*Eubacterium*] *coprostanoligenes group*, and *Family XIII AD3011 group*), two families were increased in iRBD (*Rikenellaceae* and *Akkermansiaceae*), and no genera or families were decreased in iRBD. To adjust for the environmental and dietary factors, we next examined taxonomic differences in eight pairs of iRBD patients and their spouses in our data set (Table S3a at the genus level and Table S3b at the family level in the supplemental material). Out of the nine significantly changed taxa in the unpaired analysis above, eight taxa were similarly changed in the paired analysis, although no statistical significance was observed.

10.1128/mSystems.00797-20.3 TABLE S2 (a) Genera changed in RBD in our data set. W indicates a statistical measure generated by ANCOM. *P* value was calculated by Wilcoxon rank sum test. *q* value was calculated by the Benjamini-Hochberg method. We set the significance thresholds of W > 0.6 × *N* (*N* is the number of taxa tested) and *q* value < 0.05. Significant taxa are indicated in bold. (b) Families changed in RBD in our data set. W indicates a statistical measure generated by ANCOM. *P* value was calculated by Wilcoxon rank sum test. *q* value was calculated by the Benjamini-Hochberg method. We set the significance thresholds of W > 0.6 × *N* (*N* is the number of taxa tested) and *q* value < 0.05. Significant taxa are indicated in bold. Download Table S2, DOCX file, 0.04 MB. Copyright © 2020 Nishiwaki et al. 2020 Nishiwaki et al. This content is distributed under the terms of the Creative Commons Attribution 4.0 International license.

10.1128/mSystems.00797-20.4 TABLE S3 (a) Genera changed in eight pairs of iRBD patients and their spouses in our data set. W indicates a statistical measure generated by ANCOM. *P* value was calculated by Wilcoxon signed-rank sum test. *q* value was calculated by the Benjamini-Hochberg method. Increased or decreased taxa are marked as + or −, respectively, and unchanged taxa are marked as 0. Genera which are significant in our data set including all samples are indicated in bold letters. (b) Families changed in eight pairs of iRBD patients and their spouses in our data set. W indicates a statistical measure generated by ANCOM. *P* value was calculated by Wilcoxon signed-rank sum test. *q* value was calculated by the Benjamini-Hochberg method. Increased or decreased taxa are marked as + or −, respectively, and unchanged taxa are marked as 0. Families which are significant in our data set including all samples are indicated in bold letters. Download Table S3, DOCX file, 0.03 MB. Copyright © 2020 Nishiwaki et al. 2020 Nishiwaki et al. This content is distributed under the terms of the Creative Commons Attribution 4.0 International license.

We next compared the results of iRBD-ANCOM with those of previously reported PD-ANCOM (32). Nine genera in iRBD and 24 genera in PD were increased with W > 0.5 × *N* (see Materials and Methods). Among them, seven genera (*Alistipes*, *Akkermansia*, *Ruminococcaceae UCG-005*, *Ruminococcaceae UCG-004*, *Family XIII AD3011 group*, *Ruminococcaceae_anonymous*, and *Oscillibacter*) were increased in both iRBD and PD. Similarly, two genera in iRBD and 27 genera in PD were decreased with W > 0.5 × *N*. No genera, however, were shared between iRBD and PD.

### Possible confounding factors in our data set for nine taxa that were significantly changed in iRBD in our data set.

**FIG 2:**
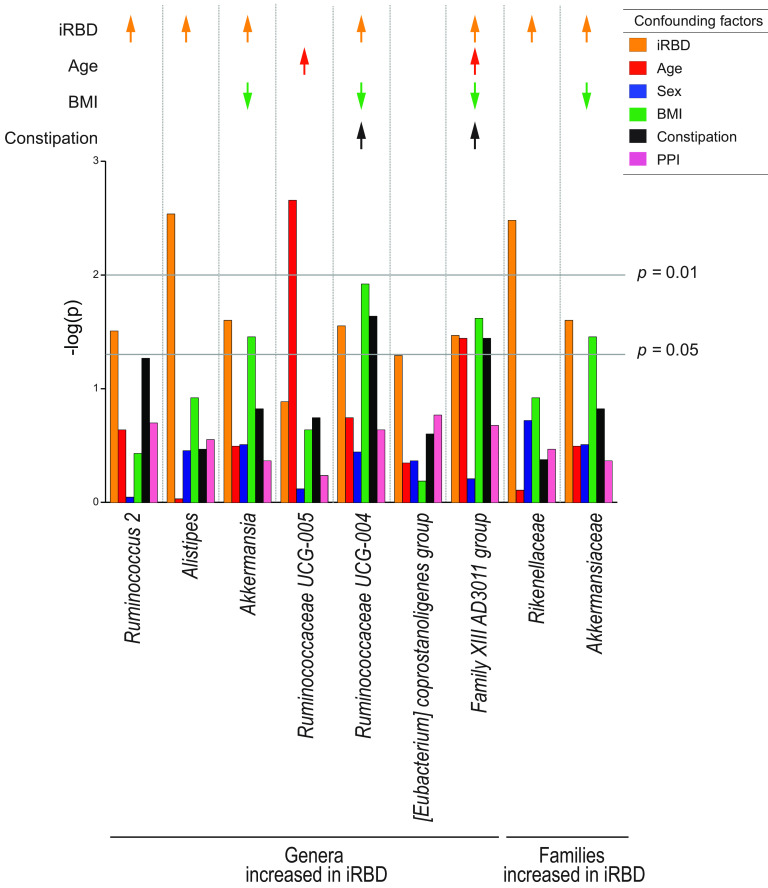
Generalized linear mixed model (GLMM) analysis to evaluate confounding factors of seven genera and two families that were significantly changed in iRBD compared to controls in our data set. The effects of iRBD, age, sex, body mass index (BMI), constipation, and PPI were individually analyzed by mutually adjusting for confounding factors by GLMM. Arrows indicate taxa that were significantly changed by iRBD (orange arrows), age (red arrows), BMI (green arrows), and constipation (black arrows) after adjusting for the other confounding factors. Upward and downward arrows indicate increased and decreased taxa, respectively. Exact *P* values are indicated in Table S4 in the supplemental material.

We next asked whether any of the nine taxonomic changes in iRBD were due to confounding factors. We thus performed Generalized Linear Mixed Model (GLMM) analysis with constipation, BMI, sex, age, and PPI. We found that five genera (*Ruminococcus 2*, *Alistipes*, *Akkermansia*, *Ruminococcaceae UCG-004*, and *Family XIII AD3011 group*) and two families (*Rikenellaceae* and *Akkermansiaceae*) were changed in iRBD after adjusting for constipation, BMI, sex, age, and PPI (Fig. 2 and bold letters in Table S4 in the supplemental material). In contrast, two genera (*Ruminococcaceae UCG-005* and *Family XIII AD3011 group*) were increased by age (Fig. 2 and underlines in Table S4 in the supplemental material). Three genera (*Akkermansia*, *Ruminococcaceae UCG-004*, and *Family XIII AD3011 group*) and one family (*Akkermansiaceae*) were decreased by BMI (Fig. 2 and underlines in Table S4 in the supplemental material). Two genera (*Ruminococcaceae UCG-004* and *Family XIII AD3011 group*) were increased by constipation (Fig. 2 and underlines in Table S4 in the supplemental material).

10.1128/mSystems.00797-20.5 TABLE S4 Exact *P* values of generalized linear mixed model (GLMM) analysis plotted in Fig. 2, as well as exact values of ANCOM analysis and Wilcoxon rank sum test. ^a^Six taxa were also significantly changed in iRBD in the meta-analysis of the Japanese and German data sets. *P* values were obtained by GLMM analysis for each confounding factor. *P* values are plotted in Fig. 2. Taxa and their *P* values that were changed in iRBD after adjusting for the effects of age, sex, BMI, constipation, and PPI are indicated in bold (also indicated by orange arrows in Fig. 2). Taxa and their *P* values that were changed by age, BMI, and constipation are underlined (also indicated by red, green, and black arrows, respectively, in Fig. 2). Note that no significantly decreased genus and family were identified in our data set. *, *P < *0.05. Download Table S4, DOCX file, 0.01 MB. Copyright © 2020 Nishiwaki et al. 2020 Nishiwaki et al. This content is distributed under the terms of the Creative Commons Attribution 4.0 International license.

### Meta-analysis of the Japanese and German data sets.

**FIG 3:**
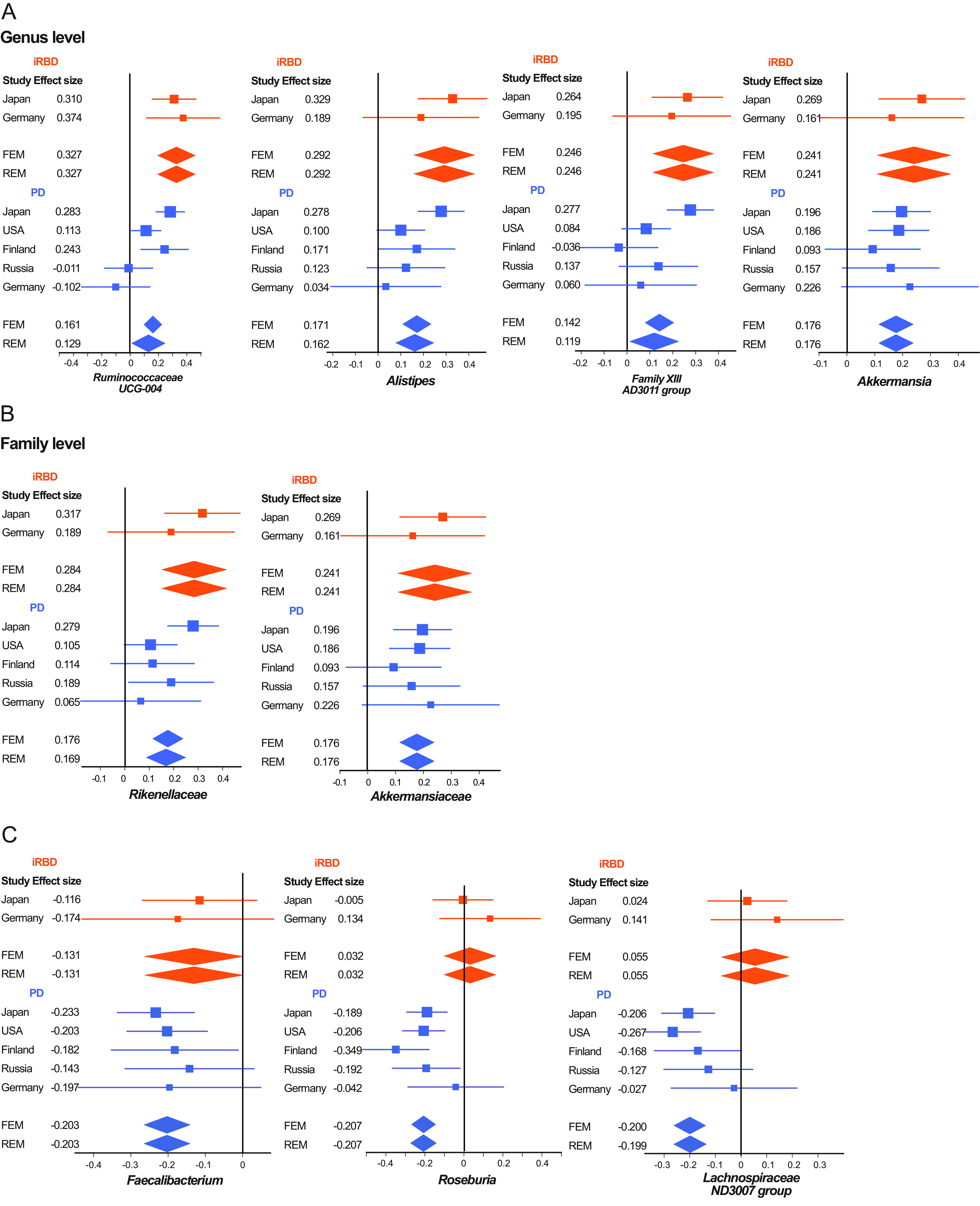
(A and B) Forest plots of four genera (A) and two families (B) that were significantly and homogenously changed in iRBD (Bonferroni-corrected *P* value < 0.05 and the homogeneity index *I*^2^ < 25%) in the Japanese and German data sets. Forest plots of PD in five data sets are also indicated in parallel. (C) Forest plots of two recognized and one putative SCFA-producing genera that were significantly and homogenously decreased in PD in five countries inour previous report (32). Forest plots of iRBD in the Japanese and German data sets are also indicated in parallel. An effect size of each data set, as well as the overall effect sizes by the fixed-effects model (FEM) and the random-effects model (REM), is indicated. Both lines and diamonds indicate 95% confidence intervals. Orange and blue symbols represent iRBD and PD, respectively. Exact statistical measures are indicated in Table S6 in the supplemental material.

Meta-analysis of gut microbiota was performed using the Japanese and German data sets (43). The effect size and relative abundance of 132 genera and 39 families are collated in Table S5a and b, respectively, in the supplemental material. Our putative criteria (*I*^2^ < 25% and *P* values of both fixed-effects model [FEM] and random-effects model [REM] after Bonferroni correction of <0.05) showed that four genera (*Ruminococcaceae UCG-004*, *Alistipes*, *Family XIII AD3011 group*, and *Akkermansia*) and two families (*Rikenellaceae* and *Akkermansiaceae*) were increased in iRBD (Fig. 3 and Table S6 in the supplemental material). These six taxa were a subset of the seven taxa that were significantly changed in iRBD after adjusting for confounding factors in our data set (Fig. 2 and bold letters in Table S4 in the supplemental material). Among the seven taxa, genus *Ruminococcus 2* was increased in Japan but not in Germany and was excluded from forest plots (Fig. 3A). Forest plots of the six taxa in iRBD in two countries along with those in PD in five countries showed that all taxa tended to be increased in PD, and the most homogenous and significant increases were observed in genus *Akkermansia* and family *Akkermansiaceae* (Fig. 3A).

10.1128/mSystems.00797-20.6 TABLE S5 (a) Effect sizes and relative abundances of all filtered genera in RBD in the meta-analysis of the Japanese and German data sets. (b) Effect sizes and relative abundances of all filtered families in RBD in the meta-analysis of the Japanese and German data sets. Download Table S5, DOCX file, 0.03 MB. Copyright © 2020 Nishiwaki et al. 2020 Nishiwaki et al. This content is distributed under the terms of the Creative Commons Attribution 4.0 International license.

10.1128/mSystems.00797-20.7 TABLE S6 Exact statistical measures of forest plots indicated in Fig. 3. FEM, fixed-effects model. REM, random-effects model. Download Table S6, DOCX file, 0.01 MB. Copyright © 2020 Nishiwaki et al. 2020 Nishiwaki et al. This content is distributed under the terms of the Creative Commons Attribution 4.0 International license.

We previously reported that two SCFA-producing genera (*Faecalibacterium* and *Roseburia*) and one putative SCFA-producing genus (*Lachnospiraceae ND3007 group*) were decreased in PD across countries (32). We assumed that genus *Lachnospiraceae ND3007 group* is a putative SCFA producer, because most genera in family *Lachnospiraceae* produce SCFA. None of the three recognized or putative SCFA-producing genera were decreased in iRBD in our meta-analysis. However, forest plots of the three genera showed that genus *Faecalibacterium* tended to be decreased in iRBD, but genera *Roseburia* and *Lachnospiraceae ND3007 group* were not (Fig. 3B).

### Relative abundances of four genera in progression of **α**-synucleinopathy.

**FIG 4:**
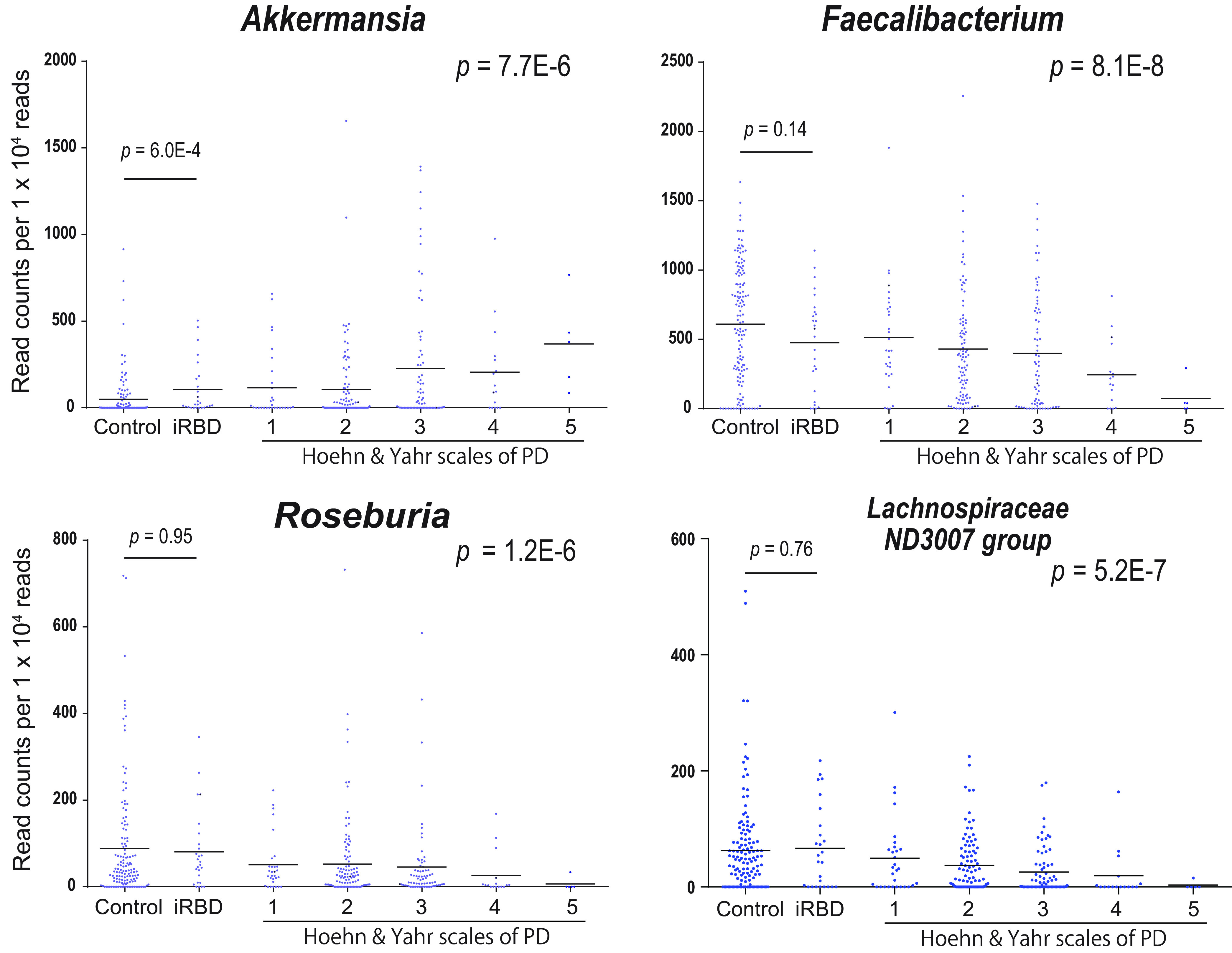
Read counts of genera *Akkermansia*, *Faecalibacterium*, *Roseburia*, and *Lachnospiraceae ND3007 group* normalized for 1 × 10^4^ reads in controls, iRBD, and Hoehn and Yahr scales 1 to 5. Bars indicate an average in each category. *P* values of Jonckheere-Terpstra trend test are shown on the right to indicate whether the genus increases or decreases monotonically. *P* values of Wilcoxon rank sum test between controls and iRBD (see Table S2a in the supplemental material) are indicated on the left. Read counts of genera *Akkermansia*, *Faecalibacterium*, and *Roseburia* in Hoehn and Yahr scales 1 to 5 were previously reported, but iRBD was not included (32).

Plots of relative abundances of genera *Akkermansia*, *Faecalibacterium*, *Roseburia*, and *Lachnospiraceae ND3007 group* in controls, iRBD, and Hoehn and Yahr scales 1 to 5 showed that genus *Akkermansia* gradually increased and genera *Faecalibacterium*, *Roseburia*, and *Lachnospiraceae ND3007 group* gradually decreased with progression of α-synucleinopathy (Fig. 4). Comparison of controls and iRBD showed that genus *Akkermansia* was significantly increased in iRBD. In contrast, genus *Faecalibacterium*, but not *Roseburia* or *Lachnospiraceae ND3007 group*, tended to be decreased in iRBD (Fig. 4).

## DISCUSSION

We analyzed gut microbiota in iRBD in our data set and meta-analyzed the Japanese and German data sets (43). We first analyzed the overall compositions of gut microbiota in controls and iRBD by PERMANOVA. Evaluation of the effects of possible confounding factors by PERMANOVA showed that the overall compositions of gut microbiota were statistically different between controls and iRBD by all three distance metrics, and the difference may or may not be affected by age but not by sex, BMI, constipation, or PPI (Table 4). PERMANOVA similarly showed that the overall compositions of gut microbiota were statistically different between iRBD and Hoehn and Yahr scale 1 by Chao and weighted UniFrac but not by unweighted UniFrac (Table 4). Again, the difference was not due to confounding factors. Weighted UniFrac takes read counts into consideration to calculate the distance so that the effects of low-abundance taxa become small, whereas low-abundance taxa have more effects on unweighted UniFrac (65). Thus, iRBD and Hoehn and Yahr scale 1 may have large differences in major taxa but not in minor taxa.

We additionally observed by PCoA that the overall compositions of gut microbiota were gradually changing in controls, iRBD, and Hoehn and Yahr scales 1 to 5 in this order (Fig. 1A). We next applied LIGER (62) to the 16S rRNA-seq analysis for the first time. LIGER, which was developed for single-cell RNA-seq analysis, enables integrative nonnegative matrix factorization (iNMF) by exploiting a topic model. Topic modeling that has been developed for text mining generally fits well to the analysis of gut microbiota (66, 67). LIGER revealed four enterotypes in controls, iRBD, and Hoehn and Yahr scales 1 to 5 in an unsupervised manner (Fig. 1B). Enterotypes were shifted with transition from control, to iRBD, to Hoehn and Yahr scales 1 to 5 (Fig. 1C). SCFA-producing genera were similarly decreased with the shift in enterotypes (see Fig. S1 in the supplemental material). We showed that genus *Akkermansia* was increased in iRBD in this communication, as well as in our previous meta-analysis of PD (32). The increase of genus *Akkermansia* in PD was also addressed in previous reports (34, 35, 38, 39, 45, 47–49) that could not be included in our previous meta-analysis of PD (32). Some reports, however, did not address the changes in *Akkermansia* in PD (31, 40–42, 44, 46). Genus *Akkermansia*, however, was not detected in factorization by LIGER. Genus *Akkermansia* was likely to be underestimated by LIGER, because multiple SCFA-producing genera were coordinately decreased in PD, whereas genus *Akkermansia* was increased alone without any accompanying genera, which reduced the chance of detecting genus *Akkermansia* by topic modeling by LIGER. Both PCoA and LIGER indicate that gut dysbiosis advances with progression of α-synucleinopathy. Alternatively, patients with α-synucleinopathy with marked gut dysbiosis may progress faster than those with mild gut dysbiosis.

Analysis of individual taxa by ANCOM and the Wilcoxon rank sum test revealed that seven genera (see Table S2a in the supplemental material) and two families (see Table S2b in the supplemental material) were increased in iRBD. Adjustment for possible confounding factors for the seven genera and two families by GLMM showed that increases of five genera (*Ruminococcus 2*, *Alistipes*, *Akkermansia*, *Ruminococcaceae UCG-004*, and *Family XIII AD3011 group*) and two families (*Rikenellaceae* and *Akkermansiaceae*) were indeed accounted for by iRBD (orange arrows in Fig. 2), although age, BMI, and constipation had additional confounding effects on three genera (*Akkermansia*, *Ruminococcaceae UCG-004*, and *Family XIII AD3011 group*) and one family (*Akkermansiaceae*). Among the five genera and two families that were increased in iRBD, only genus *Akkermansia* and family *Akkermansiaceae* were also increased in PD in our meta-analysis of five countries (32).

Meta-analysis of the Japanese and German data sets revealed that four genera (*Ruminococcaceae UCG-004*, *Alistipes*, *Family XIII AD3011 group*, and *Akkermansia*) and two families (*Rikenellaceae* and *Akkermansiaceae*) were increased in iRBD (Fig. 3A and Table S6 in the supplemental material). Among these six taxa, we previously reported that genus *Akkermansia* and family *Akkermansiaceae* were consistently increased in PD across countries (32). We found that relative abundances of genus *Akkermansia* gradually increased from iRBD to Hoehn and Yahr scales 1 to 5 (Fig. 4). Akkermansia muciniphila degrades the mucus layer of the gut (68) and erodes the mucus layer in the lack of dietary fibers (69). Indeed, intestinal permeability is increased in PD (70), and the serum lipopolysaccharide-binding protein levels are decreased in PD (31, 70). Reduced expression of a tight junction protein, occludin, in colonic biopsy specimens in PD is similarly in accordance with the reduced mucus layer (71). Increased intestinal permeability may expose the intestinal neural plexus to oxidative stress and pesticide/herbicide (72). which subsequently allows the formation of abnormal α-synuclein aggregates in the intestine. Moreover, in the presence of other gut microbiota, Akkermansia muciniphila in mouse intestine enhances differentiation of follicular T cells, which mediate humoral immunity by B cells (73, 74). A high prevalence (20%) of autoimmune diseases in female patients with RBD compared to 5% in the general population is in accordance with the *Akkermansia*-mediated increased humoral immunity (75). Similarly, RBD is sometimes associated with neuronal autoimmune diseases including narcolepsy, anti-IgLON5 disease, Kleine-Levin syndrome, multiple sclerosis, Guillain-Barré syndrome, anti-Ma2 encephalitis, LGI1 limbic encephalitis, Morvan’s syndrome, paraneoplastic cerebellar degeneration, and anti-N-methyl-d-aspartate (anti-NMDA) receptor encephalitis (76).

Meta-analysis of the Japanese and German data sets also showed that no SCFA-producing genera were decreased in iRBD (Fig. 3B and Table S6 in the supplemental material). We previously reported that three recognized and putative SCFA-producing genera (*Faecalibacterium*, *Roseburia*, and *Lachnospiraceae ND3007 group*) were consistently decreased in PD across countries (32). Although genus *Faecalibacterium* tended to be decreased in iRBD, no significance was observed (Fig. 4). In contrast, genera *Roseburia* and *Lachnospiraceae ND3007 group* were not decreased in iRBD (Fig. 4). Preservation of most of SCFA-producing bacteria in iRBD was also implicated in the LIGER analysis, which showed that both controls and iRBD were enriched in enterotype A (Fig. 1C), in which SCFA-producing bacteria were high (Fig. 1D). Major constituents of gut SCFAs, butyrate and propionate, induce anti-inflammatory regulatory T (Treg) cells by inhibiting histone deacetylase (77, 78) and by binding to G protein-coupled receptors of GPR41, GPR43, and GPR109A (79, 80). Indeed, in mouse models of PD, SCFAs may (81–83) or may not (84) have beneficial effects on PD symptoms. In addition, in another German cohort, fecal SCFA concentrations were decreased in PD (35). Our analysis suggests that reduced fecal SCFA concentrations may be a prerequisite for the development of PD but not of iRBD. Reduction of SCFA-producing bacteria culminating in the development of PD may start from genus *Faecalibacterium*. A decrease of genus *Faecalibacterium* may thus be a hallmark to predict transition from iRBD to PD. We expect that administration of SCFA and probiotics/prebiotics to increase the intestinal SCFA possibly retards the progression of α-synucleinopathy at the stage of iRBD.

## MATERIALS AND METHODS

### Patients in our data set.

All studies were approved by the Ethical Review Committees of the Nagoya University Graduate School of Medicine (approval no. 2016-0151), Iwate Medical College Hospital (H28-123), Okayama Kyokuto Hospital (kyoIR-2016002), and Fukuoka University School of Medicine (2016M027). We obtained written informed consent from all patients and controls.

We recruited 26 patients with iRBD and 137 healthy controls from four hospitals to participate in this study from September 2015 to February 2018. Among the 137 healthy controls, 8 were spouses of iRBD patients. All iRBD patients were diagnosed by International Classification of Sleep Disorders Criteria-Third Edition (57). The severity of Parkinson’s disease was determined according to Hoehn and Yahr scales 1 to 5 (85). Briefly, scale 1 indicates that a patient has only unilateral movement disability. Scale 2 indicates that a patient has bilateral movement disability but no impairment of balance. Scale 3 indicates that a patient has bilateral movement disability and impairment of balance but that his/her daily life is independent. Scale 4 indicates that a patient is severely disabled but manages to walk or stand without assistance. Scale 5 indicates that a patient is confined to bed or a wheelchair unless assisted. Subjects with diabetes mellitus, heart failure, liver cirrhosis, any malignancy, hematological diseases, and autoimmune diseases were excluded from our study. Subjects who had taken any antibiotics in the past 1 month were similarly excluded.

### DNA isolation and 16S rRNA V3-V4 sequencing in our data set.

The detailed procedures for transportation of a fecal sample from the participant’s home to the Nagoya University, freeze-drying of the fecal sample (86), and DNA isolation were described previously (32). The V3-V4 hypervariable region of the bacterial 16S rRNA gene was amplified by primer 341F, 5′-CCTACGGGNGGCWGCAG-3′ and primer 805R, 5′-GACTACHVGGGTATCTAATCC-3′. Paired-end sequencing of 300-nucleotide fragments was performed using the MiSeq reagent kit V3 on a MiSeq system (Illumina). Taxonomic analysis was performed with QIIME2 (87). Operational taxonomic units (OTUs) were generated using DADA2, and the SILVA taxonomy database release 132 (88) was used for taxonomic identification.

### Differences in demographic and clinical features, diet, and medications between controls and iRBD in our data set.

Four demographic and clinical features (age, sex, body mass index [BMI], and constipation), five medications, and 17 kinds of foods were compared between iRBD and controls in our data set using either Student’s *t* test or Fisher’s exact test. Subjects with the stool frequency of twice a week or less were defined to be constipated (89).

We examined lack of multicollinearity between iRBD, constipation, BMI, sex, age, and PPI by calculating the variance inflation factor (VIF) using the R package HH version 3.1-40. We verified that the VIFs were all less than 2, indicating that there was no multicollinearity between iRBD, constipation, BMI, sex, age, and PPI.

### Analysis of the overall gut microbiota in controls, iRBD, and Hoehn and Yahr 1 scale of PD using PERMANOVA in our data set.

Next, we analyzed the effects on the overall composition of gut microbiota of (i) iRBD versus controls and (ii) iRBD versus controls, age, sex, BMI, constipation, and PPI in our data set that was comprised of controls and iRBD using PERMANOVA (61). We similarly analyzed the effects on the overall composition of gut microbiota of (iii) iRBD versus Hoehn and Yahr scale 1 and (iv) iRBD versus Hoehn and Yahr scale 1, age, sex, BMI, constipation, and PPI in a combined data set that was comprised of our current iRBD subjects and PD subjects with Hoehn and Yahr scale 1 in our previous report (32) using PERMANOVA (61). All genera were included in this analysis. The effects were evaluated by three distance metrics of Chao (90), unweighted UniFrac (91), and weighted UniFrac (91). Chao and unweighted/weighted UniFrac distances were calculated using the R package vegan and QIIME2, respectively.

### Analysis of the overall gut microbiota in controls, iRBD, and PD using PCoA and LIGER in our data set.

For the overall analysis of gut microbiota, PD samples in our previous report (32) were included. We first performed principal-coordinate analysis (PCoA) of each subject, and the centers of gravity and standard errors in seven categories of controls, iRBD, and Hoehn and Yahr scales 1 to 5 were plotted.

We next employed the Linked Inference of Genomic Experimental Relationships (LIGER) (62), which uses integrative nonnegative matrix factorization (iNMF) for single-cell RNA-seq analysis, for unsupervised clustering of gut microbiota of controls, iRBD, and Hoehn and Yahr scales 1 to 5. LIGER enabled us to identify four enterotypes, each of which was comprised of a set of bacteria that were synchronously changed in each subject.

### Analysis of each taxon between controls and iRBD in our data set.

Taxa were filtered at the genus and family levels using the following conditions. For each taxon, we counted the number of samples in which the relative abundance of the taxon of interest was greater than 1E−4. The number of such samples should be 17 or more (more than ∼10% of all samples). We thereby chose 50 families and 168 genera.

The difference in the abundance of each taxon between iRBD and controls was analyzed by Analysis of Composition of Microbiomes (ANCOM) (63), as well as by the Wilcoxon rank sum test. ANCOM was performed on R (https://github.com/antagomir/scripts/tree/master/R/ancom). The Wilcoxon rank sum test was performed with the mannwhitneyu functionality of scipy.stat on Python 3.6.5. The threshold of W calculated in ANCOM was set to more than 0.6 × *N*, where *N* is the number of taxa. The difference in the abundance of each taxon between iRBD and controls was also analyzed by the Wilcoxon rank sum test followed by calculation of the false-discovery rate (FDR) using the Benjamini-Hochberg procedure. The FDR threshold was set to 0.05. Bacterial taxa filtered for both W and FDR were assumed to be significant.

We also analyzed the sub-data set of eight pairs of iRBD patients and their spouses by ANCOM and the Wilcoxon signed-rank sum test to adjust for the effects of diet and lifestyle.

### Possible confounding factors in our data set for nine taxa that were significantly changed in our data set.

Seven genera and two families that were identified in our data set were subjected to GLMM (Generalized Linear Mixed Model) analysis using the function “glmer.nb” of the R package lme4 by setting an option to accept taxonomic variations from subject to subject.

### Meta-analysis of the Japanese and German data sets.

Our Japanese data set was comprised of 26 iRBD patients and 137 healthy controls, whereas the German data set was comprised of 20 iRBD patients and 38 healthy controls (43). We first collated the experimental methods and demographic features (see Table S7 in the supplemental material), as well as statistical measures of sequencing depths (see Table S8 in the supplemental material) of the two data sets. The read count of each sample was all more than 10,000 in the two data sets, and no sample was excluded from our meta-analysis. For each taxon, we counted the number of samples in which the relative abundance of the taxon was more than 1E−4. We then filtered 39 families and 132 genera, in which the number of such samples was more than 10% (17/163 and 6/58) in both data sets.

10.1128/mSystems.00797-20.8 TABLE S7 Experimental methods and disease durations of the Japanese and German data sets. ^a^Mean and SD. n.a., not available. Download Table S7, DOCX file, 0.01 MB. Copyright © 2020 Nishiwaki et al. 2020 Nishiwaki et al. This content is distributed under the terms of the Creative Commons Attribution 4.0 International license.

10.1128/mSystems.00797-20.9 TABLE S8 The numbers of read counts in the Japanese and German data sets. Download Table S8, DOCX file, 0.01 MB. Copyright © 2020 Nishiwaki et al. 2020 Nishiwaki et al. This content is distributed under the terms of the Creative Commons Attribution 4.0 International license.

In the meta-analysis, we applied two criteria that we used in our previous report (32) to identify homogeneously and significantly changed taxa in the Japanese and German data sets. The two criteria were that *I*^2^, representing heterogeneity in meta-analysis, was below 25% (92) and that the *P* values after Bonferroni correction for FEM and REM were both less than 0.05.

### Data availability.

FASTQ files of our iRBD data set are available with accession number DRA009322 (https://www.ncbi.nlm.nih.gov/sra/?term=DRA009322). FASTQ files of our PD data set were previously deposited with accession number DRA009229 (https://www.ncbi.nlm.nih.gov/sra/?term=DRA009229).
