# Supplementary material for: Short-Chain Fatty Acid-Producing Gut Microbiota Is Decreased in Parkinson’s Disease but Not in Rapid-Eye-Movement Sleep Behavior Disorder
Source: mSystems. 2020 Dec 8;5(6):e00797-20. doi: 10.1128/mSystems.00797-20 (PMC7771407; doi:10.1128/mSystems.00797-20)
Supplement: TABLE S1 [file mSystems.00797-20-st001.docx]

**Supplementary Table S1. The top 10 genera with the highest loadings in the first factor by LIGER analysis**

| **Genus** | **Factor loading** |
| --- | --- |
| *Fusicatenibacter* | 33.0 |
| *Lachnospiraceae UCG-004* | 29.6 |
| *Faecalibacterium* | 27.8 |
| *Butyricicoccus* | 26.4 |
| *Anaerostipes* | 26.2 |
| *Lachnospiraceae ND3007 group* | 24.9 |
| *Bacteroides* | 24.7 |
| *Roseburia* | 24.6 |
| *Blautia* | 24.0 |
| *Lachnospira* | 23.3 |
