# Supplementary material for: Short-Chain Fatty Acid-Producing Gut Microbiota Is Decreased in Parkinson’s Disease but Not in Rapid-Eye-Movement Sleep Behavior Disorder
Source: mSystems. 2020 Dec 8;5(6):e00797-20. doi: 10.1128/mSystems.00797-20 (PMC7771407; doi:10.1128/mSystems.00797-20)
Supplement: TABLE S2 [file mSystems.00797-20-st002.docx]

**Supplementary Table S2a. Genera changed in RBD in our dataset**

| **Genus** | **W** | ***p*-value** | ***q*-value** | **Relative abundance in RBD (%)** | **Increased or decreased** |
| --- | --- | --- | --- | --- | --- |
| ***Ruminococcus 2*** | 157 | 7.7E-04 | 0.014 | 5.4 | **+** |
| ***Alistipes*** | 139 | 2.7E-05 | 4.6E-03 | 2.6 | **+** |
| ***Akkermansia*** | 130 | 6.0E-04 | 0.014 | 1.0 | **+** |
| ***Ruminococcaceae UCG-005*** | 117 | 2.2E-03 | 0.030 | 0.68 | **+** |
| ***Ruminococcaceae UCG-004*** | 112 | 7.6E-05 | 6.4E-03 | 0.27 | **+** |
| ***[Eubacterium] coprostanoligenes group*** | 109 | 1.5E-03 | 0.023 | 1.7 | **+** |
| ***Family XIII AD3011 group*** | 104 | 7.5E-04 | 0.014 | 0.27 | **+** |
| *Bacillus* | 100 | 3.8E-03 | 0.046 | 0.011 | **-** |
| *Ruminococcaceae_anonymous* | 96 | 7.3E-04 | 0.014 | 1.2 | **+** |
| *Oscillibacter* | 95 | 9.5E-04 | 0.016 | 0.42 | **+** |
| *Turicibacter* | 91 | 9.2E-03 | 0.091 | 0.020 | **-** |
| *Ruminococcaceae UCG-010* | 79 | 7.0E-04 | 0.014 | 0.092 | **+** |
| *Bifidobacterium* | 70 | 0.018 | 0.14 | 3.1 | **-** |
| *Christensenellaceae R-7 group* | 65 | 7.4E-03 | 0.077 | 0.69 | **+** |
| *Eggerthella* | 60 | 0.032 | 0.17 | 0.083 | **-** |
| *Veillonella* | 56 | 0.021 | 0.15 | 0.093 | **-** |
| *Eubacterium* | 40 | 7.7E-04 | 0.014 | 0.073 | **+** |
| *Aggregatibacter* | 40 | 0.026 | 0.16 | 0.020 | **-** |
| *Ruminococcaceae_uncultured* | 39 | 2.5E-03 | 0.032 | 0.23 | **+** |
| *Allisonella* | 38 | 0.027 | 0.16 | 6.0E-3 | **-** |
| *Peptococcaceae_uncultured* | 22 | 5.7E-04 | 0.014 | 0.015 | **+** |
| *Lachnospiraceae UCG-004* | 20 | 0.077 | 0.28 | 0.048 | **-** |
| *Prevotella 9* | 17 | 0.17 | 0.47 | 1.3 | **-** |
| *Solanum melongena (eggplant)* | 16 | 0.23 | 0.58 | 0.015 | **-** |
| *TM7 phylum sp. canine oral taxon 250* | 16 | 0.20 | 0.52 | 2.1E-03 | **-** |
| *Coprococcus 2* | 15 | 0.41 | 0.76 | 0.11 | **-** |
| *Alloprevotella* | 14 | 0.51 | 0.79 | 0.041 | **-** |
| *Enterococcus* | 14 | 0.066 | 0.26 | 0.024 | **-** |
| *Butyricicoccus* | 14 | 0.062 | 0.26 | 0.33 | **-** |
| *Lactobacillales_anonymous* | 13 | 0.44 | 0.77 | 4.5E-03 | **-** |
| *Gemella* | 12 | 0.25 | 0.62 | 5.2E-03 | **-** |
| *Merdibacter* | 12 | 0.64 | 0.89 | 7.3E-03 | **-** |
| *Mitochondria_anonymous* | 11 | 0.59 | 0.86 | 3.2E-03 | **-** |
| *Olsenella* | 10 | 0.96 | 0.98 | 0.023 | **-** |
| *Eggerthellaceae;Ambiguous_taxa* | 10 | 1.0 | 1.0 | 4.7E-03 | **+** |
| *Eggerthellaceae_uncultured* | 10 | 0.72 | 0.94 | 0.020 | **-** |
| *Tyzzerella 3* | 10 | 0.44 | 0.77 | 0.014 | **-** |
| *Candidatus Soleaferrea* | 10 | 0.72 | 0.94 | 4.4E-03 | **-** |
| *Ruminococcaceae UCG-009* | 10 | 0.36 | 0.72 | 0.011 | **+** |
| *Bacteria_anonymous* | 10 | 0.53 | 0.81 | 5.4E-03 | **-** |
| *Gordonibacter* | 9 | 0.89 | 0.98 | 0.013 | **-** |
| *Lachnospiraceae UCG-010* | 9 | 0.37 | 0.72 | 0.025 | **-** |
| *GCA-900066225* | 9 | 0.84 | 0.98 | 0.019 | **-** |
| *Coprobacillus* | 9 | 0.82 | 0.97 | 0.011 | **-** |
| *Lactonifactor* | 8 | 0.69 | 0.94 | 0.016 | **+** |
| *Anaerofustis* | 7 | 0.80 | 0.96 | 6.0E-03 | **+** |
| *Family XIII UCG-001* | 7 | 0.85 | 0.98 | 0.011 | **-** |
| *Hungatella* | 7 | 0.74 | 0.94 | 9.8E-03 | **-** |
| *Caproiciproducens* | 7 | 0.51 | 0.79 | 0.016 | **+** |
| *Phocea* | 7 | 0.98 | 0.99 | 4.3E-03 | **-** |
| *Ruminococcaceae UCG-003* | 6 | 0.64 | 0.89 | 0.067 | **-** |
| *[Clostridium] innocuum group* | 6 | 0.48 | 0.79 | 0.050 | **-** |
| *Defluviitaleaceae UCG-011* | 5 | 4.5E-03 | 0.050 | 1.3E-02 | **+** |
| *Negativibacillus* | 5 | 0.015 | 0.13 | 0.12 | **+** |
| *Rothia* | 3 | 0.72 | 0.94 | 8.7E-03 | **-** |
| *Enterorhabdus* | 3 | 0.63 | 0.89 | 0.012 | **+** |
| *Lactococcus* | 3 | 0.62 | 0.88 | 0.20 | **-** |
| *Christensenellaceae;__* | 3 | 0.043 | 0.21 | 0.019 | **+** |
| *Oscillospira* | 3 | 0.92 | 0.98 | 0.020 | **+** |
| *Firmicutes_anonymous* | 3 | 0.93 | 0.98 | 0.017 | **-** |
| *Bilophila* | 3 | 0.023 | 0.15 | 0.14 | **+** |
| *Methanobrevibacter* | 2 | 0.016 | 0.13 | 0.30 | **+** |
| *Christensenellaceae_uncultured* | 2 | 0.038 | 0.19 | 0.016 | **+** |
| *[Eubacterium] nodatum group* | 2 | 0.10 | 0.33 | 0.014 | **+** |
| *Fusicatenibacter* | 2 | 0.059 | 0.26 | 1.2 | **-** |
| *UC5-1-2E3* | 2 | 0.057 | 0.25 | 0.011 | **+** |
| *Peptococcus* | 2 | 0.038 | 0.19 | 0.10 | **+** |
| *Intestinimonas* | 2 | 0.022 | 0.15 | 0.094 | **+** |
| *Ruminiclostridium 6* | 2 | 0.017 | 0.13 | 0.34 | **+** |
| *Holdemania* | 2 | 0.031 | 0.17 | 0.040 | **+** |
| *Erysipelotrichaceae_uncultured* | 2 | 0.020 | 0.15 | 0.040 | **+** |
| *Prevotellaceae NK3B31 group* | 1 | 0.57 | 0.85 | 0.079 | **+** |
| *[Eubacterium] brachy group* | 1 | 0.071 | 0.27 | 0.060 | **+** |
| *Blautia* | 1 | 0.65 | 0.89 | 6.4 | **+** |
| *Lachnospiraceae UCG-008* | 1 | 0.94 | 0.98 | 0.020 | **-** |
| *DTU089* | 1 | 0.066 | 0.26 | 0.033 | **+** |
| *Ruminococcaceae NK4A214 group* | 1 | 0.030 | 0.17 | 0.25 | **+** |
| *UBA1819* | 1 | 0.034 | 0.18 | 0.14 | **+** |
| *Erysipelotrichaceae UCG-003* | 1 | 0.47 | 0.78 | 0.64 | **-** |
| *Erysipelotrichaceae_anonymous* | 1 | 0.59 | 0.86 | 0.017 | **+** |
| *Dialister* | 1 | 0.15 | 0.44 | 0.26 | **-** |
| *Fusobacterium* | 1 | 0.31 | 0.66 | 0.36 | **-** |
| *Actinomyces* | 0 | 0.83 | 0.98 | 0.049 | **+** |
| *Collinsella* | 0 | 0.30 | 0.65 | 1.4 | **-** |
| *Coriobacteriales Incertae Sedis_uncultured* | 0 | 0.46 | 0.77 | 0.045 | **+** |
| *Senegalimassilia* | 0 | 0.96 | 0.98 | 0.054 | **+** |
| *Slackia* | 0 | 0.29 | 0.65 | 0.065 | **+** |
| *Eggerthellaceae_anonymous* | 0 | 0.27 | 0.62 | 0.024 | **+** |
| *Bacteroides* | 0 | 0.41 | 0.76 | 20.9 | **-** |
| *Barnesiella* | 0 | 0.30 | 0.65 | 0.63 | **+** |
| *Coprobacter* | 0 | 0.55 | 0.82 | 0.12 | **+** |
| *Barnesiellaceae_uncultured* | 0 | 0.20 | 0.52 | 0.041 | **+** |
| *Butyricimonas* | 0 | 0.51 | 0.79 | 0.062 | **+** |
| *Odoribacter* | 0 | 0.085 | 0.30 | 0.28 | **+** |
| *Marinifilaceae_anonymous* | 0 | 0.19 | 0.51 | 0.028 | **+** |
| *Paraprevotella* | 0 | 0.071 | 0.27 | 0.37 | **+** |
| *Prevotella 2* | 0 | 0.095 | 0.31 | 0.58 | **+** |
| *Prevotellaceae_uncultured* | 0 | 0.37 | 0.72 | 0.046 | **+** |
| *Prevotellaceae_anonymous* | 0 | 0.47 | 0.78 | 0.16 | **+** |
| *uncultured Rikenella sp.* | 0 | 0.069 | 0.27 | 0.044 | **+** |
| *Parabacteroides* | 0 | 0.25 | 0.62 | 2.0 | **+** |
| *Bacteroidales_anonymous* | 0 | 0.77 | 0.94 | 0.078 | **+** |
| *Granulicatella* | 0 | 0.96 | 0.98 | 9.0E-03 | **-** |
| *Lactobacillus* | 0 | 0.71 | 0.94 | 1.1 | **+** |
| *Weissella* | 0 | 0.24 | 0.60 | 0.17 | **+** |
| *Streptococcus* | 0 | 0.41 | 0.76 | 3.6 | **+** |
| *Clostridium sensu stricto 1* | 0 | 0.86 | 0.98 | 0.59 | **+** |
| *Agathobacter* | 0 | 0.88 | 0.98 | 1.4 | **-** |
| *Anaerostipes* | 0 | 0.85 | 0.98 | 1.6 | **-** |
| *CAG-56* | 0 | 0.49 | 0.79 | 0.086 | **+** |
| *Coprococcus 1* | 0 | 0.77 | 0.94 | 0.22 | **+** |
| *Dorea* | 0 | 0.82 | 0.97 | 0.75 | **-** |
| *Eisenbergiella* | 0 | 0.095 | 0.31 | 0.032 | **+** |
| *GCA-900066575* | 0 | 0.29 | 0.65 | 0.055 | **+** |
| *Lachnoclostridium* | 0 | 0.16 | 0.46 | 0.56 | **+** |
| *Lachnospira* | 0 | 0.93 | 0.98 | 0.73 | **-** |
| *Lachnospiraceae FCS020 group* | 0 | 0.75 | 0.94 | 0.054 | **+** |
| *Lachnospiraceae NC2004 group* | 0 | 0.013 | 0.12 | 0.031 | **+** |
| *Lachnospiraceae ND3007 group* | 0 | 0.76 | 0.94 | 0.67 | **+** |
| *Lachnospiraceae NK4A136 group* | 0 | 0.75 | 0.94 | 0.27 | **+** |
| *Marvinbryantia* | 0 | 0.31 | 0.66 | 0.068 | **+** |
| *Moryella* | 0 | 0.45 | 0.77 | 0.022 | **+** |
| *Roseburia* | 0 | 0.95 | 0.98 | 1.3 | **-** |
| *Sellimonas* | 0 | 0.61 | 0.88 | 0.23 | **+** |
| *Tyzzerella* | 0 | 0.61 | 0.88 | 0.083 | **+** |
| *Tyzzerella 4* | 0 | 0.96 | 0.98 | 0.32 | **-** |
| *[Eubacterium] eligens group* | 0 | 0.11 | 0.35 | 0.55 | **+** |
| *[Eubacterium] fissicatena group* | 0 | 0.13 | 0.39 | 0.013 | **+** |
| *[Eubacterium] hallii group* | 0 | 0.97 | 0.98 | 1.2 | **+** |
| *[Eubacterium] ruminantium group* | 0 | 0.18 | 0.49 | 0.19 | **+** |
| *[Eubacterium] ventriosum group* | 0 | 0.76 | 0.94 | 0.23 | **+** |
| *[Eubacterium] xylanophilum group* | 0 | 0.057 | 0.25 | 0.037 | **+** |
| *[Ruminococcus] gauvreauii group* | 0 | 0.44 | 0.77 | 0.41 | **+** |
| *[Ruminococcus] gnavus group* | 0 | 0.52 | 0.80 | 0.50 | **-** |
| *[Ruminococcus] torques group* | 0 | 0.16 | 0.46 | 1.3 | **+** |
| *Lachnospiraceae_uncultured* | 0 | 0.79 | 0.96 | 0.24 | **-** |
| *Lachnospiraceae_anonymous* | 0 | 0.26 | 0.62 | 2.4 | **+** |
| *Romboutsia* | 0 | 0.35 | 0.72 | 0.030 | **+** |
| *Terrisporobacter* | 0 | 0.70 | 0.94 | 0.082 | **-** |
| *Peptostreptococcaceae_anonymous* | 0 | 0.93 | 0.98 | 1.3 | **+** |
| *Anaerotruncus* | 0 | 0.16 | 0.46 | 0.010 | **+** |
| *Faecalibacterium* | 0 | 0.14 | 0.43 | 4.8 | **-** |
| *Flavonifractor* | 0 | 0.51 | 0.79 | 0.16 | **+** |
| *Fournierella* | 0 | 0.95 | 0.98 | 0.031 | **+** |
| *Ruminiclostridium 5* | 0 | 0.27 | 0.62 | 0.50 | **+** |
| *Ruminiclostridium 9* | 0 | 0.90 | 0.98 | 0.079 | **-** |
| *Ruminococcaceae UCG-002* | 0 | 0.046 | 0.21 | 1.1 | **+** |
| *Ruminococcaceae UCG-013* | 0 | 0.77 | 0.94 | 0.34 | **-** |
| *Ruminococcaceae UCG-014* | 0 | 0.41 | 0.76 | 0.59 | **+** |
| *Ruminococcus 1* | 0 | 0.45 | 0.77 | 1.5 | **+** |
| *Subdoligranulum* | 0 | 0.95 | 0.98 | 2.3 | **-** |
| *Clostridiales_anonymous* | 0 | 0.092 | 0.31 | 0.015 | **+** |
| *Catenibacterium* | 0 | 0.72 | 0.94 | 0.26 | **-** |
| *Erysipelatoclostridium* | 0 | 0.45 | 0.77 | 0.52 | **+** |
| *Faecalitalea* | 0 | 0.29 | 0.65 | 0.26 | **+** |
| *Holdemanella* | 0 | 0.41 | 0.76 | 0.87 | **-** |
| *Acidaminococcus* | 0 | 0.41 | 0.76 | 0.057 | **-** |
| *Phascolarctobacterium* | 0 | 0.35 | 0.72 | 0.89 | **+** |
| *Megamonas* | 0 | 0.88 | 0.98 | 0.42 | **-** |
| *Megasphaera* | 0 | 0.71 | 0.94 | 0.19 | **-** |
| *Desulfovibrio* | 0 | 0.20 | 0.52 | 0.051 | **+** |
| *Parasutterella* | 0 | 0.92 | 0.98 | 0.13 | **-** |
| *Sutterella* | 0 | 0.85 | 0.98 | 0.32 | **-** |
| *Burkholderiaceae_anonymous* | 0 | 0.082 | 0.29 | 0.15 | **+** |
| *Escherichia-Shigella* | 0 | 0.46 | 0.77 | 2.2 | **-** |
| *Klebsiella* | 0 | 0.37 | 0.72 | 0.46 | **-** |
| *Enterobacteriaceae_anonymous* | 0 | 0.52 | 0.79 | 0.051 | **-** |
| *Cloacibacillus* | 0 | 0.30 | 0.65 | 0.033 | **+** |

**Supplementary Table S2b. Families changed in RBD in our dataset**

| **Family** | **W** | ***p*-value** | **q-value** | **Relative abundance in RBD (%)** | **Increased or decreased** |
| --- | --- | --- | --- | --- | --- |
| ***Rikenellaceae*** | 38 | 5.2E-05 | 2.6E-03 | 2.7 | + |
| ***Akkermansiaceae*** | 36 | 6.0E-04 | 0.010 | 1.0 | + |
| *Christensenellaceae* | 28 | 6.2E-03 | 0.031 | 0.74 | + |
| *Desulfovibrionaceae* | 28 | 8.5E-04 | 0.011 | 0.22 | + |
| *Peptococcaceae* | 26 | 1.3E-04 | 3.1E-03 | 0.12 | + |
| *Family XIII* | 23 | 1.1E-03 | 0.011 | 0.36 | + |
| *Pasteurellaceae* | 20 | 0.010 | 0.045 | 0.020 | + |
| *Bacillaceae* | 18 | 3.8E-03 | 0.024 | 0.011 | + |
| *Eubacteriaceae* | 18 | 3.0E-03 | 0.022 | 0.079 | + |
| *Bifidobacteriaceae* | 16 | 0.018 | 0.077 | 3.1 | + |
| *Ruminococcaceae* | 15 | 1.7E-03 | 0.014 | 23 | + |
| *Enterococcaceae* | 10 | 0.066 | 0.21 | 0.024 | + |
| *Atopobiaceae* | 9 | 0.26 | 0.58 | 0.026 | + |
| *Solanum melongena (eggplant)* | 9 | 0.23 | 0.58 | 0.015 | + |
| *Veillonellaceae* | 9 | 0.037 | 0.13 | 1.0 | + |
| *Family XI* | 8 | 0.25 | 0.58 | 5.2E-03 | + |
| *Lactobacillales_anonymous* | 8 | 0.44 | 0.70 | 4.5E-03 | + |
| *Mitochondria* | 8 | 0.59 | 0.79 | 3.2E-03 | + |
| *Defluviitaleaceae* | 7 | 4.5E-03 | 0.025 | 0.013 | + |
| *Lachnospiraceae* | 7 | 0.44 | 0.70 | 23 | + |
| *Saccharimonadaceae* | 7 | 0.62 | 0.80 | 3.7E-03 | + |
| *Eggerthellaceae* | 6 | 0.46 | 0.70 | 0.29 | + |
| *Erysipelotrichaceae* | 6 | 0.58 | 0.79 | 2.8 | + |
| *Micrococcaceae* | 5 | 0.72 | 0.88 | 8.7E-03 | + |
| *Fusobacteriaceae* | 5 | 0.27 | 0.58 | 0.36 | + |
| *Bacteria_anonymous_* | 5 | 0.53 | 0.76 | 5.4E-03 | + |
| *Carnobacteriaceae* | 3 | 0.96 | 0.98 | 9.0E-3 | + |
| *Synergistaceae* | 3 | 0.38 | 0.70 | 0.058 | + |
| *Methanobacteriaceae* | 2 | 0.030 | 0.11 | 0.31 | + |
| *Bacteroidales_anonymous* | 2 | 0.77 | 0.90 | 0.080 | + |
| *Firmicutes_anonymous* | 2 | 0.93 | 0.96 | 0.017 | + |
| *Bacteroidaceae* | 1 | 0.41 | 0.70 | 21 | + |
| *Marinifilaceae* | 1 | 0.05 | 0.15 | 0.38 | + |
| *Actinomycetaceae* | 0 | 0.87 | 0.95 | 0.049 | + |
| *Coriobacteriaceae* | 0 | 0.30 | 0.62 | 1.4 | + |
| *Coriobacteriales Incertae Sedis* | 0 | 0.74 | 0.88 | 0.046 | + |
| *Barnesiellaceae* | 0 | 0.35 | 0.68 | 0.79 | + |
| *Prevotellaceae* | 0 | 0.48 | 0.70 | 2.9 | + |
| *Tannerellaceae* | 0 | 0.25 | 0.58 | 2.0 | + |
| *Lactobacillaceae* | 0 | 0.72 | 0.88 | 1.1 | + |
| *Leuconostocaceae* | 0 | 0.34 | 0.68 | 0.19 | + |
| *Streptococcaceae* | 0 | 0.42 | 0.70 | 3.8 | + |
| *Clostridiaceae 1* | 0 | 0.87 | 0.95 | 0.59 | + |
| *Clostridiales vadinBB60 group* | 0 | 0.09 | 0.25 | 0.044 | + |
| *Peptostreptococcaceae* | 0 | 0.89 | 0.95 | 1.4 | + |
| *Clostridiales_anonymous* | 0 | 0.092 | 0.25 | 0.015 | + |
| *Acidaminococcaceae* | 0 | 0.84 | 0.95 | 0.95 | + |
| *Victivallaceae* | 0 | 0.60 | 0.79 | 0.032 | + |
| *Burkholderiaceae* | 0 | 0.99 | 0.99 | 0.6 | + |
| *Enterobacteriaceae* | 0 | 0.46 | 0.70 | 2.7 | + |
