# Supplementary material for: Short-Chain Fatty Acid-Producing Gut Microbiota Is Decreased in Parkinson’s Disease but Not in Rapid-Eye-Movement Sleep Behavior Disorder
Source: mSystems. 2020 Dec 8;5(6):e00797-20. doi: 10.1128/mSystems.00797-20 (PMC7771407; doi:10.1128/mSystems.00797-20)
Supplement: TABLE S3 [file mSystems.00797-20-st003.docx]

**Supplementary Table S3a. Genera** **changed in eight pairs of iRBD patients and their spouses in our dataset**

| **Genus** | ***p*-value** | ***q*-value** | **W** | **Increase or decrease** |
| --- | --- | --- | --- | --- |
| *Flavonifractor* | 0.025 | 0.58 | 0 | + |
| *Gemella* | 0.028 | 0.58 | 0 | + |
| *Agathobacter* | 0.036 | 0.58 | 146 | - |
| *Oscillibacter* | 0.036 | 0.58 | 0 | + |
| *Candidatus Soleaferrea* | 0.043 | 0.58 | 0 | + |
| *Intestinimonas* | 0.043 | 0.58 | 0 | + |
| *Ruminococcus 1* | 0.063 | 0.58 | 0 | - |
| *[Eubacterium] fissicatena group* | 0.068 | 0.58 | 0 | + |
| *Oscillospira* | 0.068 | 0.58 | 0 | - |
| *Burkholderiaceae_anonymous* | 0.068 | 0.58 | 0 | + |
| *Bacteroides* | 0.069 | 0.58 | 0 | + |
| *Ruminococcaceae UCG-013* | 0.069 | 0.58 | 0 | - |
| *Ruminococcaceae UCG-014* | 0.080 | 0.58 | 0 | - |
| *Coprobacillus* | 0.080 | 0.58 | 0 | - |
| *Lachnospiraceae_anonymous* | 0.093 | 0.58 | 0 | + |
| *Bilophila* | 0.093 | 0.58 | 0 | + |
| *Methanobrevibacter* | 0.11 | 0.58 | 0 | - |
| *Coriobacteriales Incertae Sedis_uncultured* | 0.11 | 0.58 | 0 | - |
| *Muribaculaceae_uncultured bacterium* | 0.11 | 0.58 | 0 | - |
| *Clostridiales vadinBB60 group_anonymous* | 0.11 | 0.58 | 0 | - |
| *Lachnospiraceae;D_5__CAG-56* | 0.11 | 0.58 | 0 | + |
| *Lachnospiraceae UCG-008* | 0.11 | 0.58 | 0 | - |
| *Peptococcus* | 0.11 | 0.58 | 0 | - |
| *Holdemanella* | 0.11 | 0.58 | 0 | - |
| *Turicibacter* | 0.11 | 0.58 | 0 | - |
| *Actinomyces* | 0.13 | 0.58 | 0 | + |
| *Granulicatella* | 0.13 | 0.58 | 0 | + |
| ***Family XIII AD3011 group*** | 0.13 | 0.58 | 0 | + |
| *Lachnospiraceae NK4A136 group* | 0.13 | 0.58 | 0 | - |
| *Eggerthellaceae;Ambiguous_taxa* | 0.14 | 0.58 | 0 | - |
| *Family XIII UCG-001* | 0.14 | 0.58 | 0 | - |
| *Lactonifactor* | 0.14 | 0.58 | 0 | + |
| *Fournierella* | 0.14 | 0.58 | 0 | + |
| *[Eubacterium] brachy group* | 0.16 | 0.58 | 0 | + |
| *Blautia* | 0.16 | 0.58 | 0 | + |
| *Lachnospiraceae ND3007 group* | 0.17 | 0.58 | 0 | + |
| *Klebsiella* | 0.17 | 0.58 | 0 | - |
| *Sellimonas* | 0.18 | 0.58 | 0 | + |
| *UBA1819* | 0.18 | 0.58 | 0 | + |
| *Olsenella* | 0.18 | 0.58 | 0 | - |
| *CHKCI002* | 0.18 | 0.58 | 0 | + |
| *Eggerthellaceae_uncultured* | 0.18 | 0.58 | 0 | - |
| *Prevotella 9* | 0.18 | 0.58 | 0 | - |
| *uncultured Rikenella sp.* | 0.18 | 0.58 | 0 | + |
| *Lactobacillales_anonymous* | 0.18 | 0.58 | 0 | + |
| *Defluviitaleaceae UCG-011* | 0.18 | 0.58 | 0 | + |
| *Coprococcus 2* | 0.18 | 0.58 | 0 | - |
| *Tyzzerella* | 0.18 | 0.58 | 0 | + |
| *Harryflintia* | 0.18 | 0.58 | 0 | + |
| *Pseudoflavonifractor* | 0.18 | 0.58 | 0 | - |
| *Dielma* | 0.18 | 0.58 | 0 | - |
| *Merdibacter* | 0.18 | 0.58 | 0 | - |
| *Firmicutes_anonymous* | 0.18 | 0.58 | 0 | - |
| *Mitochondria_anonymous* | 0.18 | 0.58 | 0 | + |
| *Anaerostipes* | 0.21 | 0.62 | 0 | + |
| *[Eubacterium] hallii group* | 0.21 | 0.62 | 0 | + |
| *Faecalibacterium* | 0.21 | 0.62 | 0 | - |
| *Parasutterella* | 0.21 | 0.62 | 0 | - |
| *Marinifilaceae_anonymous* | 0.22 | 0.63 | 0 | + |
| *Phocea* | 0.22 | 0.63 | 0 | - |
| *Ruminococcaceae UCG-009* | 0.22 | 0.63 | 0 | + |
| *Clostridiales_anonymous* | 0.22 | 0.63 | 0 | + |
| *Fusicatenibacter* | 0.24 | 0.63 | 0 | - |
| *Marvinbryantia* | 0.24 | 0.63 | 0 | - |
| *[Ruminococcus] gauvreauii group* | 0.24 | 0.63 | 0 | - |
| *Erysipelotrichaceae UCG-003* | 0.25 | 0.65 | 0 | - |
| *Ruminococcaceae_anonymous* | 0.26 | 0.66 | 0 | + |
| *Lactococcus* | 0.27 | 0.66 | 0 | - |
| *Solanum melongena (eggplant)* | 0.29 | 0.66 | 0 | + |
| *Enterococcus* | 0.29 | 0.66 | 0 | - |
| *Christensenella* | 0.29 | 0.66 | 0 | + |
| *Moryella* | 0.29 | 0.66 | 0 | + |
| *Acetanaerobacterium* | 0.29 | 0.66 | 0 | + |
| *Desulfovibrionaceae_uncultured* | 0.29 | 0.66 | 0 | + |
| *Enterobacteriaceae_anonymous* | 0.29 | 0.66 | 0 | - |
| *Collinsella* | 0.31 | 0.70 | 0 | - |
| *Lachnoclostridium* | 0.33 | 0.70 | 0 | + |
| *[Ruminococcus] gnavus group* | 0.33 | 0.70 | 0 | + |
| *Lachnospiraceae_uncultured* | 0.33 | 0.70 | 0 | - |
| *Subdoligranulum* | 0.33 | 0.70 | 0 | - |
| ***Akkermansia*** | 0.33 | 0.70 | 0 | + |
| *Lachnospiraceae UCG-004* | 0.35 | 0.70 | 0 | - |
| *Barnesiella* | 0.35 | 0.70 | 0 | + |
| *Christensenellaceae_uncultured* | 0.35 | 0.70 | 0 | + |
| *Faecalitalea* | 0.35 | 0.70 | 0 | + |
| *Roseburia* | 0.40 | 0.74 | 0 | + |
| *Holdemania* | 0.40 | 0.74 | 0 | + |
| *Bifidobacterium* | 0.40 | 0.74 | 0 | - |
| ***Alistipes*** | 0.40 | 0.74 | 0 | + |
| *Parabacteroides* | 0.40 | 0.74 | 0 | + |
| *Dorea* | 0.40 | 0.74 | 0 | + |
| ***Ruminococcaceae UCG-004*** | 0.40 | 0.74 | 0 | + |
| ***[Eubacterium] coprostanoligenes group*** | 0.40 | 0.74 | 0 | + |
| *Erysipelatoclostridium* | 0.40 | 0.74 | 0 | + |
| *Clostridium sensu stricto 1* | 0.46 | 0.80 | 0 | + |
| *Coprobacter* | 0.47 | 0.80 | 0 | + |
| *Prevotellaceae_anonymous* | 0.47 | 0.80 | 0 | + |
| *Weissella* | 0.47 | 0.8 | 0 | + |
| *Peptococcaceae_uncultured* | 0.47 | 0.80 | 0 | - |
| *Ruminiclostridium 6* | 0.47 | 0.80 | 0 | + |
| *Streptococcus* | 0.48 | 0.82 | 0 | + |
| *Eubacterium* | 0.50 | 0.82 | 0 | + |
| *Negativibacillus* | 0.50 | 0.82 | 0 | - |
| *Ruminococcaceae UCG-003* | 0.50 | 0.82 | 0 | + |
| *Christensenellaceae_anonymous* | 0.50 | 0.82 | 0 | + |
| *Actinobacteria_anonymous* | 0.59 | 0.84 | 0 | + |
| *Enterorhabdus* | 0.59 | 0.84 | 0 | - |
| *Prevotella 2* | 0.59 | 0.84 | 0 | + |
| *Bacteroidales_anonymous* | 0.59 | 0.84 | 0 | - |
| *Eisenbergiella* | 0.59 | 0.84 | 0 | - |
| *Erysipelotrichaceae_anonymous* | 0.59 | 0.84 | 0 | + |
| *Acidaminococcus* | 0.59 | 0.84 | 0 | - |
| *Aggregatibacter* | 0.59 | 0.84 | 0 | + |
| *Butyricimonas* | 0.61 | 0.84 | 0 | - |
| *[Eubacterium] eligens group* | 0.61 | 0.84 | 0 | - |
| *[Ruminococcus] torques group* | 0.61 | 0.84 | 0 | + |
| *Caproiciproducens* | 0.61 | 0.84 | 0 | + |
| *Ruminococcaceae NK4A214 group* | 0.61 | 0.84 | 0 | - |
| ***Ruminococcaceae UCG-005*** | 0.61 | 0.84 | 0 | + |
| *[Clostridium] innocuum group* | 0.61 | 0.84 | 0 | + |
| *Veillonella* | 0.65 | 0.84 | 0 | - |
| *Slackia* | 0.65 | 0.84 | 0 | + |
| *Marinifilaceae_uncultured organism* | 0.65 | 0.84 | 0 | + |
| *Alloprevotella* | 0.65 | 0.84 | 0 | + |
| *Prevotellaceae NK3B31 group* | 0.65 | 0.84 | 0 | - |
| *[Eubacterium] nodatum group* | 0.65 | 0.84 | 0 | - |
| *Shuttleworthia* | 0.65 | 0.84 | 0 | - |
| *UC5-1-2E3* | 0.65 | 0.84 | 0 | + |
| *Fusobacterium* | 0.65 | 0.84 | 0 | - |
| *Victivallis* | 0.65 | 0.84 | 0 | + |
| *Succinatimonas* | 0.65 | 0.84 | 0 | - |
| *Butyricicoccus* | 0.67 | 0.84 | 0 | - |
| *Eggerthellaceae_anonymous* | 0.69 | 0.84 | 0 | - |
| *Anaerotruncus* | 0.69 | 0.84 | 0 | + |
| *Megamonas* | 0.69 | 0.84 | 0 | + |
| *Bacteria_anonymous* | 0.69 | 0.84 | 0 | - |
| *Rothia* | 0.72 | 0.84 | 0 | - |
| *Senegalimassilia* | 0.72 | 0.84 | 0 | + |
| *Anaerofustis* | 0.72 | 0.84 | 0 | + |
| *GCA-900066575* | 0.72 | 0.84 | 0 | + |
| *Hungatella* | 0.72 | 0.84 | 0 | + |
| *Lachnospiraceae NC2004 group* | 0.72 | 0.84 | 0 | - |
| *Peptostreptococcaceae_anonymous* | 0.72 | 0.84 | 0 | - |
| *GCA-900066225* | 0.72 | 0.84 | 0 | - |
| *Cloacibacillus* | 0.72 | 0.84 | 0 | - |
| *Eggerthella* | 0.74 | 0.84 | 0 | + |
| *Lactobacillus* | 0.74 | 0.84 | 0 | - |
| *Christensenellaceae R-7 group* | 0.74 | 0.84 | 0 | + |
| *Lachnospiraceae FCS020 group* | 0.74 | 0.84 | 0 | - |
| *Lachnospiraceae UCG-010* | 0.74 | 0.84 | 0 | + |
| *[Eubacterium] ventriosum group* | 0.74 | 0.84 | 0 | - |
| *Lachnospira* | 0.78 | 0.88 | 0 | - |
| *Ruminiclostridium 5* | 0.78 | 0.88 | 0 | + |
| *Ruminococcaceae UCG-002* | 0.78 | 0.88 | 0 | + |
| *Tyzzerella 4* | 0.87 | 0.95 | 0 | - |
| *Ruminococcaceae_uncultured* | 0.87 | 0.95 | 0 | - |
| *Escherichia-Shigella* | 0.87 | 0.95 | 0 | + |
| *Peptostreptococcaceae_anonymous* | 0.89 | 0.95 | 0 | + |
| *Ruminiclostridium 9* | 0.89 | 0.95 | 0 | - |
| *Phascolarctobacterium* | 0.89 | 0.95 | 0 | + |
| *Sutterella* | 0.89 | 0.95 | 0 | + |
| *DTU089* | 0.89 | 0.95 | 0 | - |
| *Gordonibacter* | 0.92 | 0.96 | 0 | - |
| *Ruminococcaceae UCG-010* | 0.92 | 0.96 | 0 | - |
| ***Ruminococcus 2*** | 0.92 | 0.96 | 0 | - |
| *Odoribacter* | 1 | 1 | 0 | 0 |
| *Paraprevotella* | 1 | 1 | 0 | 0 |
| *Bacillus* | 1 | 1 | 0 | - |
| *Leuconostoc* | 1 | 1 | 0 | - |
| *Clostridiales vadinBB60 group_gut metagenome* | 1 | 1 | 0 | - |
| *Coprococcus 1* | 1 | 1 | 0 | 0 |
| *Dialister* | 1 | 1 | 0 | - |
| *Megasphaera* | 1 | 1 | 0 | + |

**Supplementary Table S3b. Families changed in eight pairs of iRBD patients and their spouses in our dataset**

| **Family** | ***p*-value** | ***q*-value** | **W** | **Increase or decrease** |
| --- | --- | --- | --- | --- |
| *Erysipelotrichaceae* | 0.036 | 0.60 | 0 | - |
| *Bacteroidaceae* | 0.069 | 0.60 | 0 | + |
| *Methanobacteriaceae* | 0.11 | 0.60 | 0 | - |
| *Family XIII* | 0.12 | 0.60 | 0 | + |
| *Actinomycetaceae* | 0.13 | 0.60 | 0 | + |
| *Carnobacteriaceae* | 0.13 | 0.60 | 0 | + |
| *Coriobacteriales Incertae Sedis* | 0.14 | 0.60 | 0 | - |
| *Peptococcaceae* | 0.14 | 0.60 | 0 | - |
| *Desulfovibrionaceae* | 0.16 | 0.60 | 0 | + |
| *Lactobacillales_anonymous* | 0.18 | 0.60 | 0 | + |
| *Defluviitaleaceae* | 0.18 | 0.60 | 0 | + |
| *Firmicutes_anonymous* | 0.18 | 0.60 | 0 | - |
| *Saccharimonadaceae* | 0.18 | 0.60 | 0 | + |
| *Mitochondria* | 0.18 | 0.60 | 0 | + |
| *Enterobacteriaceae* | 0.21 | 0.62 | 0 | - |
| *Clostridiales vadinBB60 group* | 0.22 | 0.62 | 0 | - |
| *Clostridiales_anonymous* | 0.22 | 0.62 | 0 | + |
| *Atopobiaceae* | 0.29 | 0.68 | 0 | - |
| *Solanum melongena (eggplant)* | 0.29 | 0.68 | 0 | + |
| *Enterococcaceae* | 0.29 | 0.68 | 0 | - |
| *Coriobacteriaceae* | 0.31 | 0.68 | 0 | - |
| *Eubacteriaceae* | 0.33 | 0.68 | 0 | + |
| ***Akkermansiaceae*** | 0.33 | 0.68 | 0 | + |
| *Barnesiellaceae* | 0.35 | 0.69 | 0 | + |
| *Bifidobacteriaceae* | 0.40 | 0.69 | 0 | - |
| *Marinifilaceae* | 0.40 | 0.69 | 0 | + |
| ***Rikenellaceae*** | 0.40 | 0.69 | 0 | + |
| *Tannerellaceae* | 0.40 | 0.69 | 0 | + |
| *Prevotellaceae* | 0.46 | 0.73 | 0 | - |
| *Clostridiaceae 1* | 0.46 | 0.73 | 0 | + |
| *Streptococcaceae* | 0.48 | 0.73 | 0 | + |
| *Burkholderiaceae* | 0.48 | 0.73 | 0 | + |
| *Bacteroidales_anonymous* | 0.59 | 0.86 | 0 | - |
| *Fusobacteriaceae* | 0.65 | 0.86 | 0 | - |
| *Ruminococcaceae* | 0.67 | 0.86 | 0 | - |
| *Acidaminococcaceae* | 0.67 | 0.86 | 0 | - |
| *Leuconostocaceae* | 0.69 | 0.86 | 0 | + |
| *Bacteria_anonymous* | 0.69 | 0.86 | 0 | - |
| *Micrococcaceae* | 0.72 | 0.86 | 0 | - |
| *Synergistaceae* | 0.74 | 0.86 | 0 | - |
| *Lactobacillaceae* | 0.74 | 0.86 | 0 | - |
| *Christensenellaceae* | 0.74 | 0.86 | 0 | + |
| *_Lachnospiraceae* | 0.78 | 0.86 | 0 | + |
| *Veillonellaceae* | 0.89 | 0.97 | 0 | - |
| *Pasteurellaceae* | 0.89 | 0.97 | 0 | - |
| *Eggerthellaceae* | 1 | 1 | 0 | 0 |
| *Bacillaceae* | 1 | 1 | 0 | - |
| *Peptostreptococcaceae* | 1 | 1 | 0 | 0 |
| *Victivallaceae* | 1 | 1 | 0 | - |
