# Supplementary material for: Short-Chain Fatty Acid-Producing Gut Microbiota Is Decreased in Parkinson’s Disease but Not in Rapid-Eye-Movement Sleep Behavior Disorder
Source: mSystems. 2020 Dec 8;5(6):e00797-20. doi: 10.1128/mSystems.00797-20 (PMC7771407; doi:10.1128/mSystems.00797-20)
Supplement: TABLE S4 [file mSystems.00797-20-st004.docx]

**Supplementary table S4. Exact *p*-values of generalized linear mixed model (GLMM) analysis plotted in Fig. 2, as well as exact values of ANCOM analysis and Wilcoxon rank sum test**

|  | **W by ANCOM** | ***P*-value by Wilcoxon rank sum test** | ***P*-value by GLMM** | | | | | |
| --- | --- | --- | --- | --- | --- | --- | --- | --- |
|  |  |  | **iRBD** | **Age** | **Sex** | **BMI** | **Constipation** | **PPI** |
| **Increased at the genus level** |  |  |  |  |  |  |  |  |
| ***Ruminococcus 2*** | 157 | ^*^7.7E-4 | **^*^0.031** | 0.23 | 0.90 | 0.37 | 0.054 | 0.2 |
| ***Alistipes*^a^** | 139 | ^*^2.7E-5 | **^*^2.9E-3** | 0.93 | 0.35 | 0.12 | 0.34 | 0.28 |
| ***Akkermansia*^a^** | 130 | ^*^6.0E-4 | **^*^0.025** | 0.32 | 0.31 | ^*^0.035 | 0.15 | 0.43 |
| *Ruminococcaceae UCG-005* | 117 | ^*^2.2E-3 | 0.13 | ^*^2.2E-3 | 0.76 | 0.23 | 0.18 | 0.58 |
| ***Ruminococcaceae UCG-004^a^*** | 112 | ^*^7.6E-5 | **^*^0.028** | 0.18 | 0.36 | ^*^0.012 | ^*^0.023 | 0.23 |
| *[Eubacterium] coprostanoligenes group* | 109 | ^*^1.5E-3 | 0.051 | 0.45 | 0.43 | 0.65 | 0.25 | 0.17 |
| ***Family XIII AD3011 group*^a^** | 104 | ^*^7.5E-4 | **^*^0.034** | ^*^0.036 | 0.62 | ^*^0.024 | ^*^0.036 | 0.21 |
| **Increased at the family level** |  |  |  |  |  |  |  |  |
| ***Rikenellaceae*^a^** | 38 | ^*^5.2E-5 | **^*^3.3E-3** | 0.78 | 0.19 | 0.12 | 0.42 | 0.34 |
| ***Akkermansiaceae*^a^** | 36 | ^*^6.0E-4 | **^*^0.025** | 0.32 | 0.31 | ^*^0.035 | 0.15 | 0.43 |
