# Supplementary material for: Short-Chain Fatty Acid-Producing Gut Microbiota Is Decreased in Parkinson’s Disease but Not in Rapid-Eye-Movement Sleep Behavior Disorder
Source: mSystems. 2020 Dec 8;5(6):e00797-20. doi: 10.1128/mSystems.00797-20 (PMC7771407; doi:10.1128/mSystems.00797-20)
Supplement: TABLE S5 [file mSystems.00797-20-st005.docx]

**Supplementary Table S5a. Effect sizes and relative abundances of all filtered genera in RBD in the meta-analysis of the Japanese and German datasets**

| **Genus** | **Effect size** | **Relative abundance in RBD (%)** |
| --- | --- | --- |
| *Ruminococcaceae UCG-004* | 0.33 | 0.18 |
| *Alistipes* | 0.29 | 2.7 |
| *Family XIII AD3011 group* | 0.25 | 0.24 |
| *Akkermansia* | 0.24 | 2.0 |
| *UBA1819* | 0.24 | 0.14 |
| *[Eubacterium] coprostanoligenes group* | 0.23 | 1.4 |
| *Ruminococcaceae_uncultured* | 0.21 | 0.26 |
| *Oscillibacter* | 0.21 | 0.39 |
| *Bacillus* | -0.20 | 8.6E-03 |
| *Ruminococcus 2* | 0.20 | 3.8 |
| *Eisenbergiella* | 0.20 | 0.028 |
| *Ruminococcaceae NK4A214 group* | 0.19 | 0.37 |
| *Anaerotruncus* | 0.19 | 0.022 |
| *Ruminococcaceae_anonymous* | 0.19 | 2.2 |
| *Ruminococcaceae UCG-010* | 0.19 | 0.12 |
| *Negativibacillus* | 0.19 | 0.18 |
| *Holdemania* | 0.18 | 0.035 |
| *Christensenellaceae R-7 group* | 0.18 | 2.1 |
| *Lachnospiraceae NC2004 group* | 0.18 | 0.070 |
| *Turicibacter* | -0.18 | 0.025 |
| *UC5-1-2E3* | 0.17 | 0.011 |
| *Prevotella 9* | -0.17 | 1.7 |
| *Ruminococcaceae UCG-005* | 0.17 | 0.55 |
| *Ruminiclostridium 6* | 0.17 | 0.57 |
| *DTU089* | 0.16 | 0.027 |
| *[Ruminococcus] torques group* | 0.16 | 1.3 |
| *[Eubacterium] xylanophilum group* | 0.16 | 0.093 |
| *Bifidobacterium* | -0.16 | 2.4 |
| *[Eubacterium] nodatum group* | 0.14 | 0.010 |
| *Faecalibacterium* | -0.13 | 6.9 |
| *Ruminococcaceae UCG-002* | 0.13 | 1.1 |
| *Dialister* | -0.1 | 0.17 |
| *Lachnoclostridium* | 0.13 | 0.61 |
| *Lachnospiraceae UCG-004* | -0.13 | 0.14 |
| *Marinifilaceae_anonymous* | 0.13 | 0.054 |
| *Ruminiclostridium 5* | 0.13 | 0.35 |
| *Burkholderiaceae_anonymous* | 0.12 | 0.62 |
| *Lachnospiraceae_anonymous* | 0.12 | 4.1 |
| *Parabacteroides* | 0.11 | 2.1 |
| *Coprococcus 2* | -0.11 | 0.24 |
| *Clostridiales_anonymous* | 0.11 | 0.27 |
| *Marvinbryantia* | 0.11 | 0.10 |
| *Methanobrevibacter* | 0.11 | 0.21 |
| *Butyricimonas* | 0.11 | 0.14 |
| *Butyricicoccus* | -0.10 | 0.29 |
| *Barnesiella* | 0.10 | 0.68 |
| *Erysipelatoclostridium* | 0.10 | 0.33 |
| *Flavonifractor* | 0.10 | 0.16 |
| *Intestinimonas* | 0.091 | 0.079 |
| *Odoribacter* | 0.090 | 0.31 |
| *Erysipelotrichaceae_uncultured* | 0.089 | 0.027 |
| *Erysipelotrichaceae_anonymous* | 0.089 | 0.037 |
| *Slackia* | 0.085 | 0.053 |
| *Ruminococcaceae UCG-014* | 0.081 | 0.48 |
| *Eggerthellaceae_anonymous* | 0.079 | 0.067 |
| *Sellimonas* | 0.078 | 0.14 |
| *Prevotella 2* | 0.077 | 0.37 |
| *Barnesiellaceae_uncultured* | 0.077 | 0.083 |
| *TM7 phylum sp. canine oral taxon 250* | -0.073 | 2.5E-03 |
| *[Eubacterium] eligens group* | 0.072 | 0.53 |
| *Romboutsia* | 0.072 | 0.018 |
| *Coprococcus 1* | 0.072 | 0.23 |
| *Paraprevotella* | 0.071 | 0.27 |
| *Megasphaera* | -0.071 | 0.11 |
| *Lachnospiraceae UCG-008* | -0.066 | 0.042 |
| *Blautia* | 0.066 | 4.7 |
| *Eggerthellaceae_uncultured* | -0.066 | 0.043 |
| *Candidatus Soleaferrea* | -0.064 | 4.6E-03 |
| *Catenibacterium* | -0.064 | 0.28 |
| *Merdibacter* | -0.063 | 6.7E-03 |
| *Ruminococcaceae UCG-003* | -0.062 | 0.12 |
| *Clostridium sensu stricto 1* | -0.062 | 0.40 |
| *Streptococcus* | 0.059 | 2.2 |
| *Coprobacter* | 0.059 | 0.087 |
| *Lachnospira* | -0.058 | 0.66 |
| *Cloacibacillus* | 0.056 | 0.034 |
| *Fusicatenibacter* | -0.056 | 1.1 |
| *Lachnospiraceae ND3007 group* | 0.055 | 0.76 |
| *GCA-900066225* | 0.054 | 0.013 |
| *Collinsella* | -0.051 | 1.1 |
| *Tyzzerella* | 0.048 | 0.061 |
| *Lachnospiraceae UCG-010* | -0.048 | 0.039 |
| *Erysipelotrichaceae UCG-003* | -0.048 | 0.48 |
| *Eggerthella* | -0.048 | 0.091 |
| *Coriobacteriales Incertae Sedis_ncultured* | 0.047 | 0.040 |
| *[Ruminococcus] gnavus group* | 0.047 | 0.35 |
| *[Clostridium] innocuum group* | 0.047 | 0.049 |
| *[Eubacterium] ruminantium group* | 0.046 | 0.38 |
| *Faecalitalea* | 0.046 | 0.17 |
| *Dorea* | -0.043 | 0.63 |
| *Phocea* | 0.043 | 5.0E-03 |
| *Klebsiella* | -0.043 | 0.28 |
| *[Ruminococcus] gauvreauii group* | 0.043 | 0.38 |
| *Peptostreptococcaceae_anonymous* | -0.043 | 0.75 |
| *Desulfovibrio* | 0.040 | 0.29 |
| *Ruminococcaceae UCG-009* | 0.039 | 1.1E-02 |
| *Phascolarctobacterium* | 0.032 | 0.86 |
| *Ruminococcaceae UCG-013* | 0.032 | 0.24 |
| *Agathobacter* | -0.032 | 1.7 |
| *Roseburia* | 0.032 | 1.4 |
| *[Eubacterium] hallii group* | 0.031 | 1.2 |
| *Coprobacillus* | 0.030 | 0.019 |
| *Prevotellaceae_anonymous* | -0.028 | 0.27 |
| *Lactococcus* | 0.027 | 0.12 |
| *Fusobacterium* | -0.027 | 0.20 |
| *[Eubacterium] ventriosum group* | 0.026 | 0.14 |
| *Ruminiclostridium 9* | -0.024 | 0.11 |
| *Alloprevotella* | -0.024 | 0.12 |
| *Actinomyces* | 0.023 | 0.033 |
| *Gordonibacter* | 0.023 | 0.012 |
| *Bacteria_anonymous* | -0.023 | 0.076 |
| *Prevotellaceae_uncultured* | 0.023 | 0.073 |
| *Lactobacillus* | 0.021 | 0.91 |
| *Moryella* | 0.021 | 0.019 |
| *Tyzzerella 3* | -0.019 | 0.040 |
| *Bacteroidales_anonymous* | 0.018 | 0.42 |
| *Terrisporobacter* | -0.017 | 0.062 |
| *Ruminococcus 1* | -0.016 | 1.2 |
| *Lachnospiraceae FCS020 group* | 0.012 | 0.077 |
| *Firmicutes_anonymous* | 0.012 | 0.26 |
| *Senegalimassilia* | -7.7E-03 | 0.05 |
| *CAG-56* | 7.0E-03 | 0.11 |
| *Holdemanella* | 6.8E-03 | 0.75 |
| *Lachnospiraceae NK4A136 group* | -6.3E-03 | 0.47 |
| *Oscillospira* | 5.5E-03 | 0.045 |
| *Anaerostipes* | -4.7E-03 | 1.3 |
| *Olsenella* | -4.6E-03 | 0.023 |
| *Bacteroides* | 4.2E-03 | 19 |
| *Lachnospiraceae_uncultured* | -4.1E-03 | 0.17 |
| *Enterobacteriaceae;__* | 7.2E-04 | 0.03 |
| *Subdoligranulum* | 7.1E-04 | 2.2 |
| *GCA-900066575* | 9.5E-05 | 0.059 |

**Supplementary Table S5b. Effect sizes and relative abundances of all filtered families in RBD in the meta-analysis of the Japanese and German datasets**

| **Family** | **Effect size** | **Relative abundance (%)** |
| --- | --- | --- |
| *Rikenellaceae* | 0.28 | 2.8 |
| *Akkermansiaceae* | 0.24 | 2.0 |
| *Family XIII* | 0.21 | 0.31 |
| *Bacillaceae* | -0.20 | 8.6E-03 |
| *Veillonellaceae* | -0.19 | 0.63 |
| *Christensenellaceae* | 0.18 | 2.1 |
| *Pasteurellaceae* | -0.18 | 0.020 |
| *Marinifilaceae* | 0.17 | 0.52 |
| *Ruminococcaceae* | 0.17 | 24 |
| *Desulfovibrionaceae* | 0.16 | 0.40 |
| *Bifidobacteriaceae* | -0.16 | 2.4 |
| *Clostridiales vadinBB60 group* | 0.12 | 0.080 |
| *Clostridiales;__* | 0.11 | 0.27 |
| *Tannerellaceae* | 0.10 | 2.1 |
| *Barnesiellaceae* | 0.087 | 0.86 |
| *Atopobiaceae* | -0.087 | 0.034 |
| *Methanobacteriaceae* | 0.083 | 0.21 |
| *Eggerthellaceae* | -0.075 | 0.34 |
| *Burkholderiaceae* | -0.069 | 1.0 |
| *Clostridiaceae 1* | -0.062 | 0.40 |
| *Streptococcaceae* | 0.061 | 2.3 |
| *Prevotellaceae* | -0.056 | 3.1 |
| *Coriobacteriaceae* | -0.051 | 1.1 |
| *Synergistaceae* | 0.050 | 0.053 |
| *Fusobacteriaceae* | -0.032 | 0.20 |
| *Victivallaceae* | 0.029 | 0.050 |
| *Peptostreptococcaceae* | -0.027 | 1.0 |
| *Coriobacteriales Incertae Sedis* | 0.026 | 0.043 |
| *Lachnospiraceae* | 0.025 | 24 |
| *Bacteria* | -0.023 | 0.076 |
| *Saccharimonadaceae* | -0.021 | 3.6E-03 |
| *Lactobacillaceae* | 0.019 | 0.91 |
| *Bacteroidales_anonymous* | 0.018 | 0.42 |
| *Actinomycetaceae* | 0.016 | 0.033 |
| *Firmicutes* | 0.012 | 0.26 |
| *Enterobacteriaceae* | -6.7E-03 | 2.1 |
| *Acidaminococcaceae* | -6.0E-03 | 1.1 |
| *Erysipelotrichaceae* | 4.4E-03 | 2.3 |
| *Bacteroidaceae* | 4.2E-03 | 19 |
