# Supplementary material for: Short-Chain Fatty Acid-Producing Gut Microbiota Is Decreased in Parkinson’s Disease but Not in Rapid-Eye-Movement Sleep Behavior Disorder
Source: mSystems. 2020 Dec 8;5(6):e00797-20. doi: 10.1128/mSystems.00797-20 (PMC7771407; doi:10.1128/mSystems.00797-20)
Supplement: TABLE S6 [file mSystems.00797-20-st006.docx]

**Supplementary table S6. Exact statistical measures of forest plots indicated in Fig. 3**

|  | **iRBD** | | | | **PD** | | | |
| --- | --- | --- | --- | --- | --- | --- | --- | --- |
|  | ***p*-value (FEM)** | ***p*-value (REM)** | ***I^2^* (%)** | **Relative abundance (%)** | ***p*-value (FEM)** | ***p*-value (REM)** | ***I^2^* (%)** | **Relative abundance (%)** |
| **A. Significantly increased in iRBD in two datasets at the genus level (plotted in Fig. 3A)** | | | | | | | | |
| *Ruminococcaceae UCG-004* | 1.20E-06 | 1.20E-06 | 0 | 0.27 | 2.90E-07 | 0.029 | 74 | 0.13 |
| *Alistipes* | 1.40E-05 | 1.40E-05 | 0 | 2.6 | 4.80E-08 | 1.20E-04 | 45 | 2.7 |
| *Family XIII AD3011 group* | 2.50E-04 | 2.50E-04 | 0 | 0.27 | 6.30E-06 | 0.026 | 67 | 0.13 |
| *Akkermansia* | 3.50E-04 | 3.50E-04 | 0 | 1 | 1.90E-08 | 1.90E-08 | 0 | 2.7 |
| **B. Significantly increased in iRBD in two datasets at the family level (plotted in Fig. 3B)** | | | | | | | | |
| *Rikenellaceae* | 2.50E-05 | 2.50E-05 | 0 | 2.7 | 2.00E-08 | 3.80E-05 | 41 | 2.8 |
| *Akkermansiaceae* | 3.50E-04 | 3.50E-04 | 0 | 1 | 1.00E-08 | 1.90E-08 | 0 | 2.7 |
| **C. Significantly decreased in PD at the genus level (plotted in Fig. 3C)** | | | | | | | | |
| *Faecalibacterium* | 0.052 | 0.052 | 0 | 6.9 | 4.40E-11 | 7.20E-10 | 0 | 4.8 |
| *Roseburia* | 0.64 | 0.64 | 0 | 1.4 | 1.00E-11 | 1.00E-11 | 12 | 0.57 |
| *Lachnospiraceae ND3007 group* | 0.41 | 0.41 | 0 | 0.76 | 2.00E-10 | 6.50E-10 | 4.5 | 0.2 |
