# Supplementary material for: Short-Chain Fatty Acid-Producing Gut Microbiota Is Decreased in Parkinson’s Disease but Not in Rapid-Eye-Movement Sleep Behavior Disorder
Source: mSystems. 2020 Dec 8;5(6):e00797-20. doi: 10.1128/mSystems.00797-20 (PMC7771407; doi:10.1128/mSystems.00797-20)
Supplement: TABLE S7 [file mSystems.00797-20-st007.docx]

**Supplementary Table S7. Experimental methods and disease durations of the Japanese and German datasets**

| **Country** | **Transportation temperature** | **Storage method** | **Stool DNA stabilizer** | **Sequencing** | **Primers** | **Disease duration (years)^a^** |
| --- | --- | --- | --- | --- | --- | --- |
| Japan | 0 °C | Freeze dry | no | 16S rRNA V3-4 | 341F/805R | 6.4 ± 4.8  (Max 20, Min 0.1) |
| Germany | On dry ice | −80 °C | no | 16S rRNA V4 | 515F/805R | n.a. |
