# Supplementary material for: Short-Chain Fatty Acid-Producing Gut Microbiota Is Decreased in Parkinson’s Disease but Not in Rapid-Eye-Movement Sleep Behavior Disorder
Source: mSystems. 2020 Dec 8;5(6):e00797-20. doi: 10.1128/mSystems.00797-20 (PMC7771407; doi:10.1128/mSystems.00797-20)
Supplement: TABLE S8 [file mSystems.00797-20-st008.docx]

**Supplementary Table S8. The numbers of read counts in the Japanese and German datasets**

(x 1000 read counts)

| **Country (sample size)** | **Average** | **SD** | **Median** | **Max** | **Min** |
| --- | --- | --- | --- | --- | --- |
| Japan (163) | 53.5 | 18.7 | 50.8 | 98.0 | 13.8 |
| Germany (58) | 155.7 | 39.5 | 164.8 | 261.4 | 63.7 |
